# Encapsulation of the septal cell wall protects *Streptococcus pneumoniae* from its major peptidoglycan hydrolase and host defenses

**DOI:** 10.1371/journal.ppat.1010516

**Published:** 2022-06-22

**Authors:** Joana Figueiredo, Mafalda Xavier Henriques, Maria João Catalão, Sara Pinheiro, Ana Rita Narciso, Francisco Mesquita, Bruno Manuel Saraiva, Madalena Carido, Didier Cabanes, Mariana Gomes Pinho, Sérgio Raposo Filipe

**Affiliations:** 1 Laboratory of Bacterial Cell Surfaces and Pathogenesis, UCIBIO-Applied Molecular Biosciences Unit, Department of Life Sciences, NOVA School of Science and Technology, Universidade Nova de Lisboa, Monte da Caparica, Portugal; 2 Laboratory of Bacterial Cell Surfaces and Pathogenesis, Instituto de Tecnologia Química e Biológica, Universidade Nova de Lisboa, Oeiras, Portugal; 3 Associate Laboratory i4HB—Institute for Health and Bioeconomy, School of Science and Technology, NOVA University Lisbon, Monte da Caparica, Portugal; 4 Host-Pathogen Interaction Unit, Faculty of Pharmacy, Research Institute for Medicines, iMed-ULisboa, Universidade de Lisboa, Lisboa, Portugal; 5 Group of Molecular Microbiology, Instituto de Investigação e Inovação em Saúde (i3S), Universidade do Porto, Porto, Portugal; 6 Laboratory of Bacterial Cell Biology, Instituto de Tecnologia Química e Biológica António Xavier, Universidade Nova de Lisboa, Oeiras, Portugal; The University of Alabama at Birmingham, UNITED STATES

## Abstract

Synthesis of the capsular polysaccharide, a major virulence factor for many pathogenic bacteria, is required for bacterial survival within the infected host. In *Streptococcus pneumoniae*, Wze, an autophosphorylating tyrosine kinase, and Wzd, a membrane protein required for Wze autophosphorylation, co-localize at the division septum and guarantee the presence of capsule at this subcellular location. To determine how bacteria regulate capsule synthesis, we studied pneumococcal proteins that interact with Wzd and Wze using bacterial two hybrid assays and fluorescence microscopy. We found that Wzd interacts with Wzg, the putative ligase that attaches capsule to the bacterial cell wall, and recruits it to the septal area. This interaction required residue V56 of Wzd and both the transmembrane regions and DNA-PPF domain of Wzg. When compared to the wild type, Wzd null pneumococci lack capsule at midcell, bind the peptidoglycan hydrolase LytA better and are more susceptible to LytA-induced lysis, and are less virulent in a zebrafish embryo infection model. In this manuscript, we propose that the Wzd/Wze pair guarantees full encapsulation of pneumococcal bacteria by recruiting Wzg to the division septum, ensuring that capsule attachment is coordinated with peptidoglycan synthesis. Impairing the encapsulation process, at localized subcellular sites, may facilitate elimination of bacteria by strategies that target the pneumococcal peptidoglycan.

## Author summary

During their cell cycle, Gram-positive bacteria can be frequently found surrounded by a cellular envelope, which includes glycopolymers such as peptidoglycan, wall teichoic acids or capsular polysaccharides, whose composition is adjusted to the external insults they may find. The clarification of how bacteria tune synthesis of these three macromolecules, which act as defensive layers, ensuring efficient enclosing of bacteria and their protection from the surrounding medium, will permit a better understanding of how bacteria propagate and may pave the way to the intelligent design of anti-infective strategies. In this manuscript, we have determined how Wzg, a candidate for the *Streptococcus pneumoniae* ligase that attaches capsular polysaccharides to the bacterial cell wall, is directed to the division septum to ensure the full encapsulation of bacteria. Perturbation of this process result in bacteria with reduced protection from external peptidoglycan binding receptors (such as the pneumococcal major hydrolase) and impaired in their ability to survive within the infected host.

## Introduction

*Streptococcus pneumoniae* is an important Gram-positive bacterial pathogen, which can colonize asymptomatically the nasopharynx of healthy individuals [1] or spread to other sites of the human host causing disease such as otitis media, pneumonia, or meningitis [2]. The capsule, a polysaccharide that, together with wall teichoic acids, decorates the peptidoglycan (PGN) macromolecule at the bacterial cell surface [3], is crucial for the ability of pneumococcus to cause invasive disease, since less encapsulated or non-encapsulated strains are less virulent or avirulent [4–6]. The capsule surrounds the surface of bacteria and acts as a shield that confers protection against the host’s immune system [7–9]. A tight control of the amount of capsular polysaccharide (CPS) expressed or linked to the cell surface is expected to occur during the infection process, as a thick capsule may be disadvantageous during the colonization stage of infection, by preventing the exposure of bacterial molecules required for surface adhesion [2].

To date, more than one hundred capsular serotypes have been identified in pneumococcal bacteria [10–13]. The genes encoding proteins involved in CPS synthesis and regulation are, in most cases, located in the same region of the *S*. *pneumoniae* chromosome: the *cps* operon (S1 Fig, data adapted from Bentley et al. [14]). Most proteins encoded within the *cps* operon are serotype specific including those involved in the assembly of the repeating CPS unit, responsible for the transport of the CPS repeating unit from the inner to the outer face of the plasma membrane, involved in the polymerization of different repeating units and in the assembly of a mature CPS. However, the proteins encoded by the first four genes at the 5’ end of the *cps* operon are highly conserved between serotypes and are proposed to be involved in the regulation of CPS synthesis [14]. The first gene in the *cps* operon encodes Wzg, also known as CpsA, an enzyme that belongs to the LCP (**L**ytR–**C**ps2A –**P**sr) protein family. The initial observation that LytR protein produced by *B*. *subtilis* was involved in transcription regulation, has led to the proposal that Wzg (named Cps19A) had a similar role in serotype 19A encapsulated pneumococci [15,16]. However, a more recent study from Kawai and colleagues provided strong evidence that LCP proteins are in fact phosphotransferase enzymes implicated in anchoring anionic cell wall polymers, such as teichoic acids and capsular polysaccharides, to peptidoglycan [17]. Several reports suggest that Wzg may play a role in the attachment of CPS to the bacterial cell surface. Crystallography studies by Eberhardt and colleagues support the proposed phosphotransferase activity for the serotype 2 Wzg protein [18]. Pneumococcal *wzg* mutant strains produce less total and cell wall associated CPS [19]. In addition, a pneumococcal *wzg-lytR* double mutant, which has a strongly distorted morphology, releases more capsule material to the supernatant when compared to the single *wzg* mutant and the parental strain [18]. Recently, a *lytR* single mutant was shown to be impaired in the retention of both CPS and teichoic acids [20]. However, the observed reduction in cell wall CPS does not directly imply a specific defect in attachment dependent on the activity of Wzg. Furthermore the three LCP homologs may have semi-redundant roles in the attachment of CPS and teichoic acids precursor chains to peptidoglycan [18].

Besides *wzg*, the conserved region of the *cps* operon contains *wzh*, *wzd*, and *wze* (S1 Fig), which encode three proteins that are considered to be part of a phosphoregulatory system required to control the synthesis of the capsule and its attachment to the cell wall [19]. Wze, also known as CpsD, is an autophosphorylating tyrosine kinase that belongs to the Bacterial Tyrosine Kinase family [21]. It has the conserved motifs characteristic of this protein family: a Walker A ATP binding motif and a C-terminal tyrosine cluster [22]. Wzd, also known as CpsC, is a membrane protein required for the autophosphorylation of Wze [23]. Wzh, also known as CpsB, is a phosphotyrosine protein phosphatase of the PHP family that dephosphorylates Wze [24]. For years, the role of tyrosine phosphorylation in capsular polysaccharide synthesis has been the subject of debate, with different reports presenting conflicting results, either supporting that Wze phosphorylation reduces capsule synthesis [22] or that it positively regulates CPS synthesis [25,26]. More recently, it has been reported that neither Wze phosphorylation, nor Wzh phosphatase activity, are determinants of capsule levels and factors outside the capsule locus have been suggested to play a role in the production of capsule [27].

Previously, we identified a novel role for Wzd and Wze as spatial regulators of capsule synthesis [28]. Wzd and Wze localize at the division septum in a manner that is dependent on the presence of both proteins, but independent of all other proteins encoded in the *cps* operon. In the absence of Wzd or Wze, capsule is synthesized, and covalently attached to the cell wall through phosphodiester bonds, given that it can only be removed through treatment with hydrofluoric acid [28], but it is absent from the division septum, the site where cell wall synthesis occurs during division. Therefore, we proposed that Wzd and Wze function as spatial regulators of capsule synthesis, ensuring that it occurs at the division septum, possibly to conceal newly synthesized cell wall [28]. A second role for the Wzd/Wze complex is to recruit the polysaccharide polymerase Wzy to the division site [29]. This suggests that CPS is produced only at the division septum by a single machinery [29]. In a *wzd* or *wze* null mutant, capsule is absent from the septum, but present in the rest of the cell surface, probably due to the observed delocalization of the CPS synthesis machinery [29]. This shows that these mutants are still able to export and attach polysaccharides to the cell surface.

Currently, the model for regulation of *S*. *pneumoniae* capsular polysaccharide synthesis proposes that when non-phosphorylated Wze interacts with Wzd, both proteins localize to the division septum [28,29]. Upon interaction with Wzd, Wze undergoes a conformational switch, promoting ATP binding and allowing autophosphorylation of its C-terminal tyrosine cluster [22,23]. Wze can then be dephosphorylated by the phosphatase Wzh and the cyclic phosphorylation/dephosphorylation of Wze could regulate the activity of the capsule assembly machinery [23,30]. Likely, Wzd and Wze capture the CPS synthesis machinery at the division site and trigger CPS export by the flippase Wzx, followed by polymerization by Wzy and anchoring to the peptidoglycan mesh by the phosphotransferase Wzg [14,17,18,29].

In this work, we examined the mechanism by which septal Wzd/Wze ensure CPS attachment to the septal cell wall. We demonstrate that this process is mediated by the recruitment of the Wzg ligase. When Wzg is not recruited to the division septum, there is an absence of septal CPS that results in bacteria with septal cell wall more exposed to external PGN hydrolases or host PGN receptors.

## Results

### The CPS-cell wall ligase Wzg interacts with Wzd

We have previously found that Wzd and Wze act as spatial regulators of the *S*. *pneumoniae* capsule synthesis, to ensure that capsule is present at midcell, at the newly produced cell surface [28]. However, the mechanism mediating this regulation has remained unclear. We hypothesized that the Wzd/Wze complex either activates or recruits particular proteins of the CPS machinery synthesis to the division site [28].

In order to screen for possible interactions between Wzd or Wze proteins from *S*. *pneumoniae* ATCC6314 strain and other proteins involved in the synthesis of CPS, we have used a Bacterial Two-Hybrid (BTH) assay [31], which is based on the expression, in an *Escherichia coli* strain deficient in endogenous adenylate cyclase, of two inactive fragments of the catalytic domain of *Bordetella pertussis* adenylate cyclase, T25 and T18. When fused to interacting polypeptides, these fragments are brought together, activate the synthesis of cAMP and consequently restore the ability of the *E*. *coli* mutant strain to ferment lactose or maltose.

**Fig 1:**
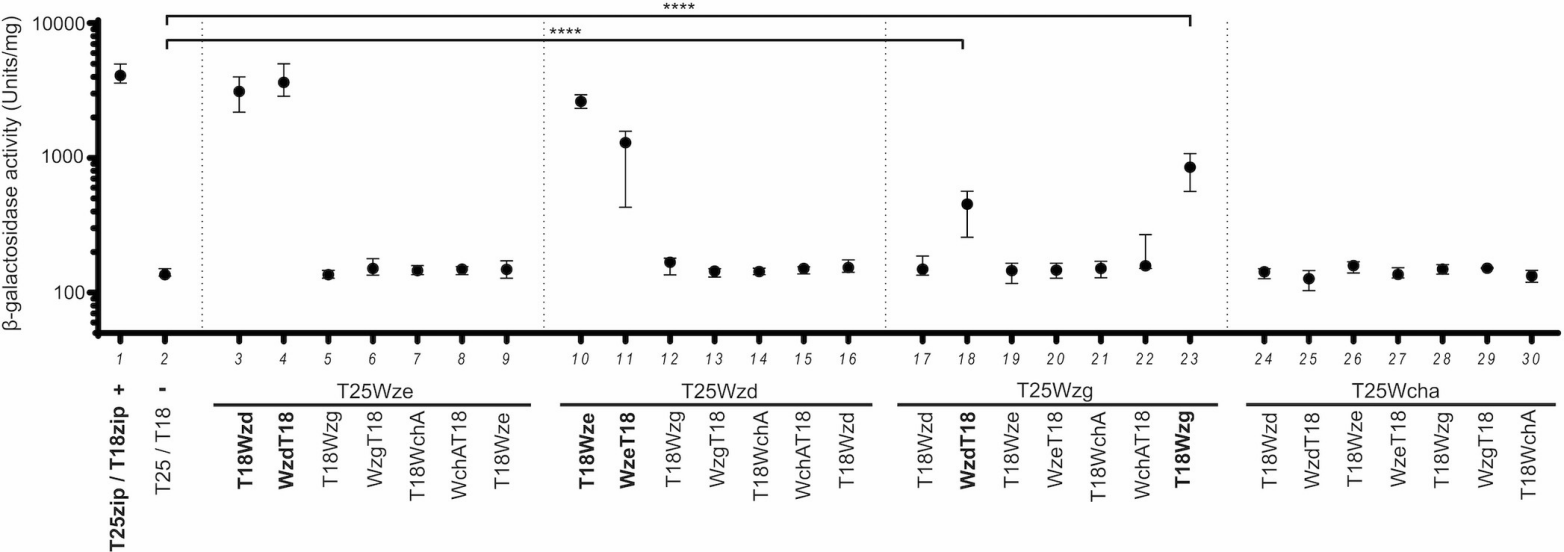
Wzg, a putative CPS-cell wall ligase, interacts with Wzd and with itself. Interactions between Wze, Wzd, Wzg and WchA were tested using Bacterial Two-Hybrid *E*. *coli* system [31]. β-galactosidase activity of cells expressing putative interaction partners was measured in cell extracts in at least three independent replicates. Black circles indicate median values and brackets show the 25% and 75% percentiles. Positive control (+): *E*. *coli* expressing T18 and T25 fragments linked to leucine zipper domains (zip) that can dimerize; Negative control (-): *E*. *coli* expressing untagged T18 and T25 fragments. Interactions were detected between T25-Wzg/Wzd-T18 and T25-Wzg/T18-Wzg. ****P ≤ 0.0001. As previously described [28] combinations of plasmids expressing Wzd and Wze indicate that these proteins interact.

We screened for possible interactions between Wzd or Wze proteins from *S*. *pneumoniae* ATCC6314 strain, which produces a serotype 14 capsular polysaccharide, and two candidate proteins that could have a role in the control of the total amount of capsule present at the bacterial surface: WchA, the glycosyl transferase that links the first glucose residue of the capsule repeating unit to the lipid anchor present in the membrane of pneumococci [32]; and Wzg, the putative CPS-cell wall ligase that has been proposed to be involved in the linkage of assembled capsule molecules [18], which have been exported across the bacterial membrane but are still linked to a lipid anchor, to the peptidoglycan macromolecule, preventing the release of capsule to the surrounding medium [17]. An interaction was found only between Wzd and Wzg (Fig 1, column 18), which was dependent on the T25/T18 tag that was linked to the interacting proteins (Fig 1, column 12), while no interactions were found between Wzd/Wze and WchA. Wzg interactions were observed when the T25-tag was fused to the N-terminal of the protein (Fig 1), but not when it was fused to the C-terminal (S2 Fig). Wzg was also found to interact with itself (Fig 1, column 23). In accordance with what has been previously reported [28,29], we were also able to detect an interaction between Wzd and Wze (Fig 1, columns 3–4 and 10–11).

### Wzg septal localization is dependent on the presence of Wzd/Wze proteins

Given that Wzg interacted with Wzd in the BTH assay, and that Wzd has been shown to localize at the septum of encapsulated *S*. *pneumoniae* cells [28], we determined whether the septal localization of Wzg in dividing pneumococcal bacteria was dependent on the expression, or localization, of Wzd. For that, we constructed pneumococcal strains expressing a N-terminal fusion of Wzg linked to iGFP, an improved version of GFP for in vivo localization of proteins in *S*. *pneumoniae* [33]. The iGFP-Wzg fusion is functional as it can produce CPS at the surface of bacteria with the chromosomal *wzg* copy deleted from the *cps* locus (S3A Fig).

**Fig 2:**
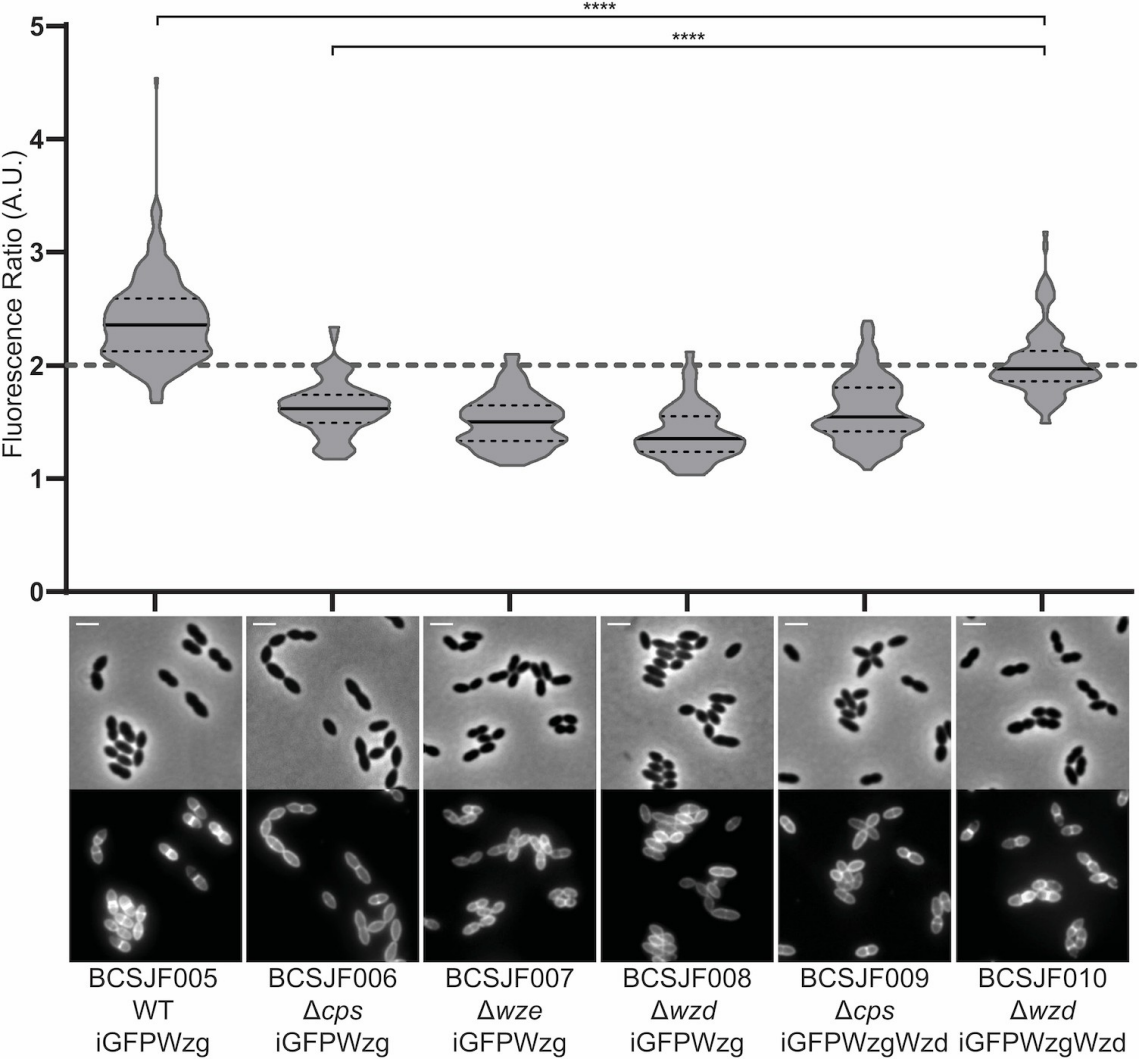
Septal localization of Wzg is dependent on the expression of Wzd/Wze. Graph shows the ratio of iGFP-Wzg fluorescence measured at the septum versus the peripheral wall in the *S*. *pneumoniae* wild-type encapsulated strain (BCSJF005, n = 194), in the *cps* null mutant (BCSJF006, n = 121), in the *wze* null mutant (BCSJF007, n = 106), in the *wzd* null mutant (BCSJF008, n = 107) and in the *cps* and *wzd* null mutants expressing Wzd from a constitutive promoter (BCSJF009, n = 112, and BCSJF010, n = 109, respectively). Enrichment of Wzg at the septum is only observed when Wzd is expressed and is localized at the division septum. Solid lines indicate median, and dashed lines indicate 25% and 75% percentiles. Representative phase contrast and fluorescence microscopy images of each strain are shown below the graph. ****P ≤ 0.0001. Scale bar, 2 μm.

A pneumococcal plasmid expressing iGFP-Wzg was transformed into the wild-type ATCC6314 encapsulated strain (generating strain BCSJF005), into its unencapsulated isogenic strain that lacks the entire *cps* operon (generating strain BCSJF006), into the ATCC6314 *wze* null mutant in which Wzd is unable to localize at the septum [28] (generating strain BCSJF007), and into the ATCC6314 *wzd* null mutant (generating strain BCSJF008). When the resulting strains were observed by fluorescence microscopy, the fluorescent signal from iGFP-Wzg was found to be more enriched at the septal region of encapsulated BCSJF005 cells, than in cells from the unencapsulated strain that lack the *cps* operon or from the *wze* and *wzd* null mutant strains (Fig 2). In these, iGFP-Wzg was spread throughout the cell surface, presumably dispersed in the cell membrane, in accordance with the presence of three predicted transmembrane spanning domains in Wzg protein [34]. A quantitative analysis of these results was done by determining the ratio of the fluorescence signal at the septum versus the peripheral membrane (FR), as described in the Materials and Methods section. Notice that the septum contains two membranes, so only FR values higher than ~2 indicate preferential septal localization [35,36]. The encapsulated strain had a median FR value of 2.4, while strains lacking the *cps* operon, or the *wze* or *wzd* genes, had median FR values below 2 (1.6, 1.5 and 1.4, respectively, Fig 2), suggesting that Wzd is required for septal localization of Wzg. In agreement, complementation of the *wzd* null mutant with plasmid encoded Wzd (strain BCSJF010) caused an increase in recruitment of Wzg to the septum (median FR value of 2.0, Fig 2). It is possible that full complementation was not achieved because Wzd in the plasmid is under the control of a constitutive promoter, instead of the *cps* promoter. Septal enrichment of Wzg was not recovered upon complementation of the unencapsulated strain BCSJF009 with plasmid encoded Wzd (median FR value of 1.5, Fig 2). In this strain Wzd is present but it is delocalized due to the absence of Wze [28]. Therefore, septal Wzd appears to be required for the recruitment of Wzg to the septum, as expected if a physical interaction between the two proteins was responsible for Wzg localization. Similar data was obtained using a CFP fusion to Wzg (S3 Fig) showing that septal Wzg enrichment is independent of the fluorescent tag used.

In contrast to Wzg, WchA localization does not show septal enrichment. Expression of a functional derivative of WchA-CFP, which supports capsule production (S4 Fig, panel A), in an encapsulated or in a *cps* null mutant strain resulted in bacteria with a fluorescence signal dispersed throughout the membrane (S4B Fig).

Together, these results indicate that Wzg localizes at the division septum of pneumococcal cells, in a process that requires the correct expression and septal localization of Wzd/Wze.

### The transmembrane and DNA-PPF domains of Wzg are required for its interaction with the membrane protein Wzd and with itself

**Fig 3:**
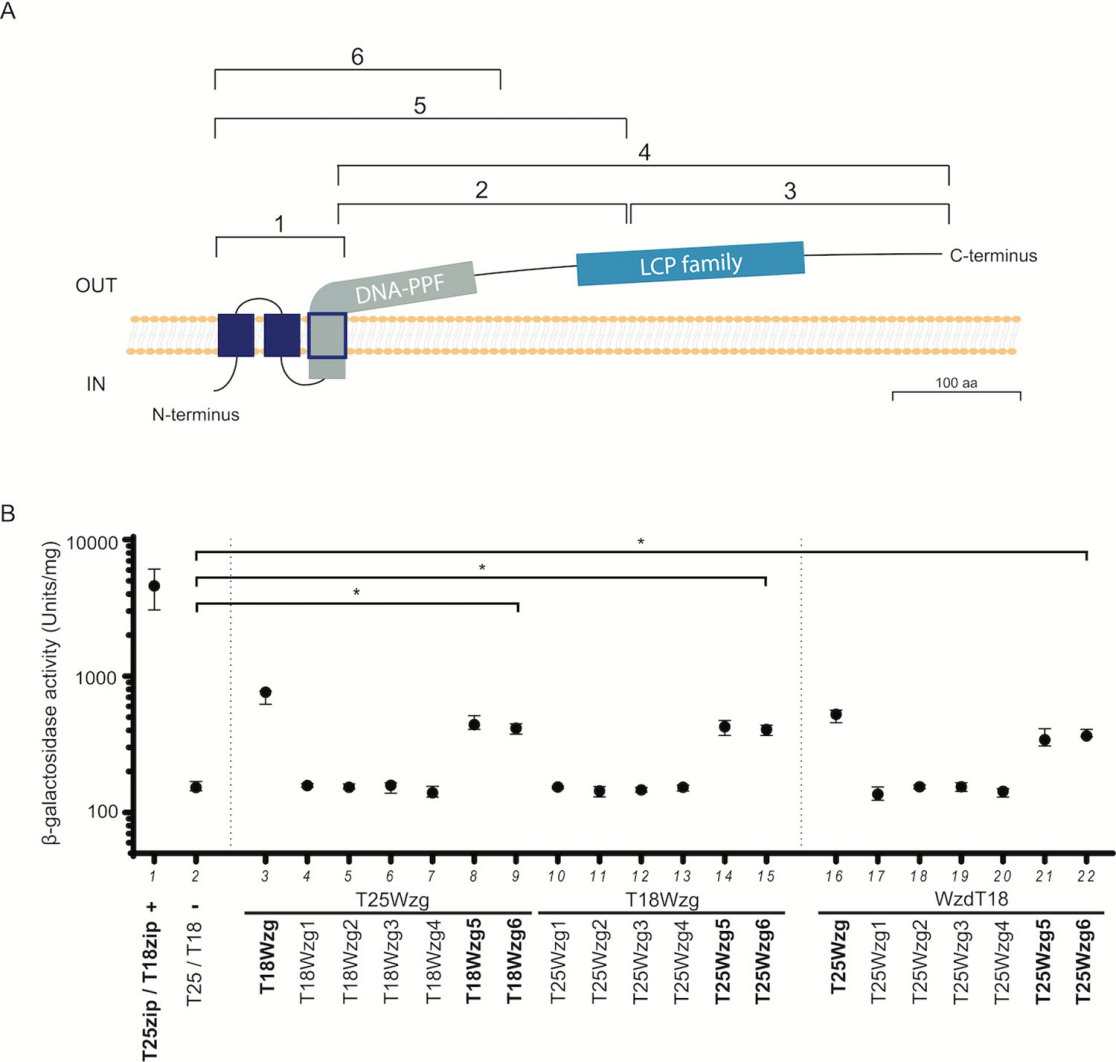
The transmembrane and DNA-PPF domains of Wzg are required for its self-interaction and interaction with Wzd. A) Scheme indicating the 6 different regions of Wzg tested for interaction with Wzd by BTH. B) Interactions between Wzg and the Wzg1-6 constructs were analyzed by BTH. Black circles indicate median values and brackets show the 25% and 75% percentiles of β-galactosidase activity measured in cell extracts of at least three independent replicates. Positive control (+): *E*. *coli* expressing T18 and T25 fragments linked to leucine zipper domains that can dimerize; Negative control (-): *E*. *coli* expressing untagged T18 and T25 fragments. *P ≤ 0.05. Only the Wzg5 and Wzg6 constructs, which include the three transmembrane domains and the DNA-PPF domain, interact with full-length Wzg and with Wzd.

Wzg is constituted by a small intracellular domain at its N-terminus, followed by three membrane spanning domains and a large extracellular C-terminal domain (Fig 3A). This large extracellular protein region contains an accessory domain, named DNA-PPF, present in some proteins of the LCP family, and the LytR-CpsA-PsR domain (Fig 3A), considered to be the core catalytic domain, present in all proteins of this family [34].

To determine which regions are involved in Wzg interactions, we used again a BTH assay. For this purpose, 6 different fragments of Wzg, shown in Fig 3A, were tested, showing that the three transmembrane domains and the first 120 amino acids of the extracellular domain (containing the accessory DNA-PPF domain), are required for self-interaction of Wzg monomers (Fig 3B, see columns 8/9 and 14/15) and for the interaction between Wzg and Wzd (Fig 3B, see columns 21 and 22). These results suggest that Wzd recruits Wzg to the pneumococcal division septum through interaction with the membrane anchored accessory DNA-PPF domain.

### Wzd V56 residue is critical for interaction with, and septal recruitment of, Wzg

Having determined the region of the Wzg ligase required for interaction with Wzd, we then enquired which region of the Wzd regulator was involved in this interaction.

**Fig 4:**
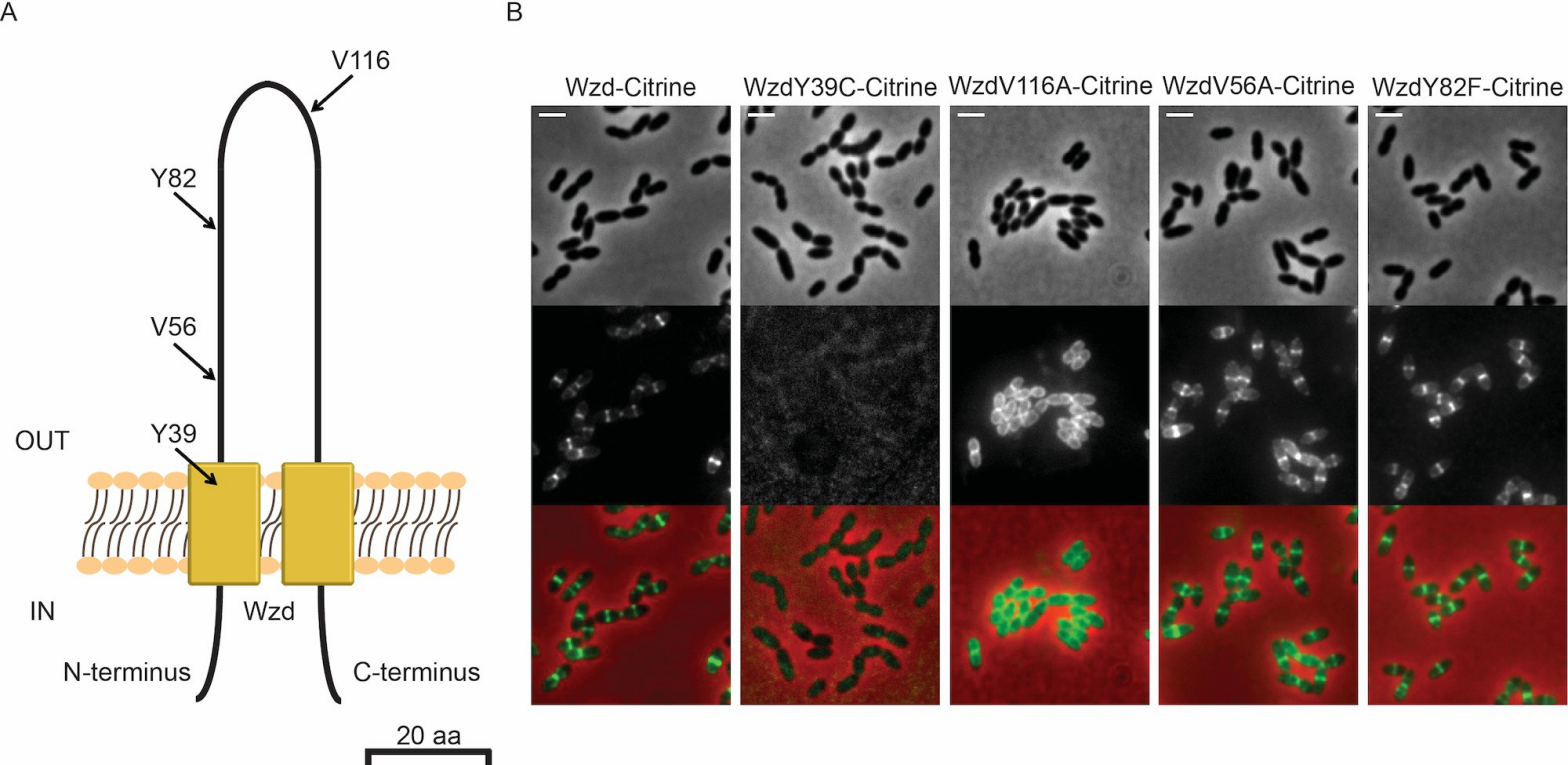
Point mutations in Wzd affect its localization. A) Scheme of Wzd protein indicating the localization, in the extracellular loop, of four mutations that were associated with a mucoid colony phenotype [19]. B) Localization of Citrine fluorescent derivatives of Wzd single-residue mutants expressed in a *wzd* null mutant strain. Strain BCSMH022, expressing Wzd-Citrine was used as control. V56A and Y82F mutations did not interfere with the ability of Wzd to localize at the division septum upon interaction with Wze. Representative phase contrast (top panels, for visualization of bacteria), fluorescence microscopy (middle panels, for detection of the capsule associated with the bacterial cell surface) and overlay (bottom panels) images of each strain are shown. Scale bar, 2 μm.

Morona and colleagues have previously described that specific point mutations in Wzd, when in the presence of a mutated Wzh phosphatase, are associated with deficient attachment of capsular polysaccharide (CPS) to the cell wall [19]. Mutants bearing these alterations present a mucoid phenotype, in which total CPS levels are similar to the wild-type strain, but levels of CPS attached to the cell wall are much lower [19]. These observations indicate that the specific residues that were identified may be important for the ligation of CPS to the cell wall, but how this takes place has remained elusive. We hypothesized that these residues could be involved in Wzd interaction with the Wzg ligase. To test this hypothesis, and as the reported mucoid mutants carried other mutations beside those reported in Wzd, we first tested whether four of the reported mutations, namely Y39C, V56A, Y82F and V116A [19], impaired expression, or septal localization, of Wzd. All these residues are located in the extracellular loop of Wzd (Fig 4A), which could be responsible for its interaction with and recruitment of Wzg. We therefore expressed fluorescent derivatives of the four Wzd mutant alleles in a *wzd* null mutant and determined their septal localization by fluorescence microscopy (Fig 4B). The ability of a mutated Wzd to localize at the division septum indicates that the protein is able to interact with Wze and may still regulate the synthesis of the pneumococcal CPS. The Y39C mutation may affect expression or stability of the Wzd protein, as no fluorescent cells were observed in the strain expressing WzdY39C. The V116A mutation impaired correct localization of Wzd at the septum, which became spread throughout the entire cell membrane. This mislocalization could be due to lack of interaction with Wze, which is required for septal localization of Wzd, or due to misfolding, or other alterations, that prevented the presence of an active Wzd. In contrast, mutations V56A and Y82F did not impair correct septal Wzd localization (Fig 4B). We then asked whether WzdV56A and WzdY82F mutants were altered in their ability to interact with Wzg and to recruit the capsule ligase to the division septum of pneumococci.

**Fig 5:**
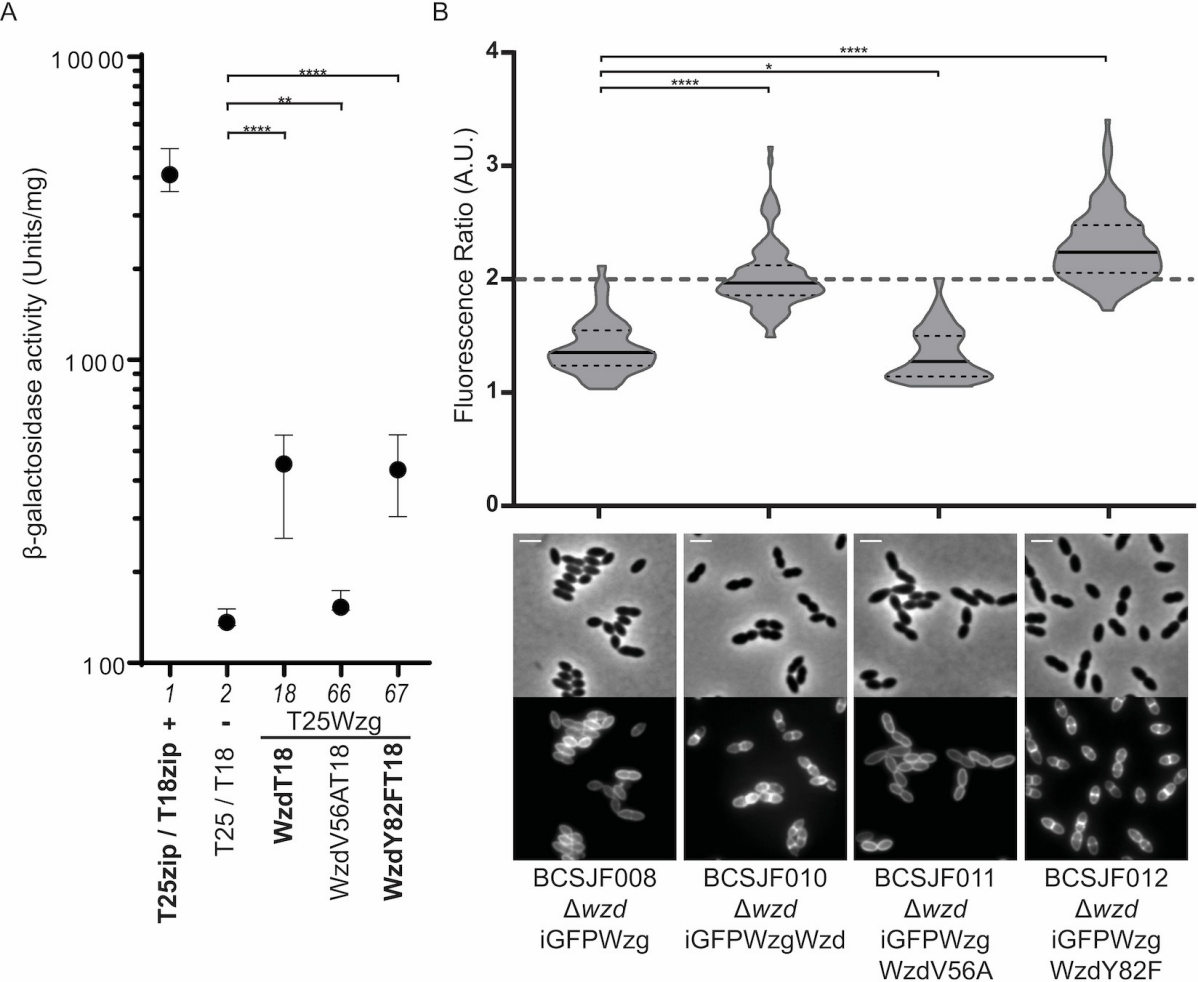
The valine residue at position 56 of Wzd is required for its interaction with Wzg, and for the recruitment of Wzg to the division septum. A) Interaction between Wzg and Wzd, WzdV56A and WzdY82F point mutants was tested by BTH. Black circles indicate median values and brackets show the 25% and 75% percentiles of β-galactosidase activity measured in cell extracts of at least three independent replicates. Positive control (+): *E*. *coli* expressing T18 and T25 fragments linked to leucine zipper domains (zip) that can dimerize; Negative control (-): *E*. *coli* expressing untagged T18 and T25 fragments. WzdV56A-T18 lost the ability to interact with T25-Wzg. B) Graph shows the ratio of iGFP-Wzg fluorescence measured at the septum versus the peripheral wall in the *S*. *pneumoniae wzd* null mutant strain (BCSJF008, n = 107) and in the *wzd* null mutant strain expressing Wzd or Wzd point mutants from a constitutive promoter (non-mutated Wzd strain BCSJF010, n = 109, mutant WzdV56A strain BCSJF011, n = 106, and mutant WzdY82F strain BCSJF012, n = 128). WzdY82F, but not WzdV56A, can recruit Wzg to the division septum of pneumococcal bacteria. *P ≤ 0.05, **P ≤ 0.01, ****P ≤ 0.0001.

In the BTH assay WzdV56A, but not WzdY82F, lost the ability to interact with Wzg (Fig 5A, columns 66 and 67) indicating that V56 is critical for Wzd/Wzg interaction. We next tested whether WzdV56A was also unable to interact with Wzg in pneumococcal cells, leading to Wzg mis-localization. For this purpose, we co-expressed, in a *wzd* null mutant, the fusion protein iGFP-Wzg with either Wzd (strain BCSJF010), WzdY82F (strain BCSJF012) or WzdV56A (strain BCSJF011). As described above, when Wzd was constitutively expressed from a plasmid in a strain lacking *wzd*, iGFP-Wzg was able to localize at the division septum (median FR of 2.0). Similarly, WzdY82F was able to recruit iGFP-Wzg to the septum (median FR of 2.2) (Fig 5B). On the contrary, complementation with WzdV56A, did not recruit iGFP-Wzg to the division septum, as the fluorescent signal was distributed over the cell membrane (median FR of 1.3), similarly to the non-complemented strain BCSJF008 (median FR of 1.4) (Fig 5B).

### Wzg recruitment to the septum by Wzd is required for the presence of capsular polysaccharide at midcell

**Fig 6:**
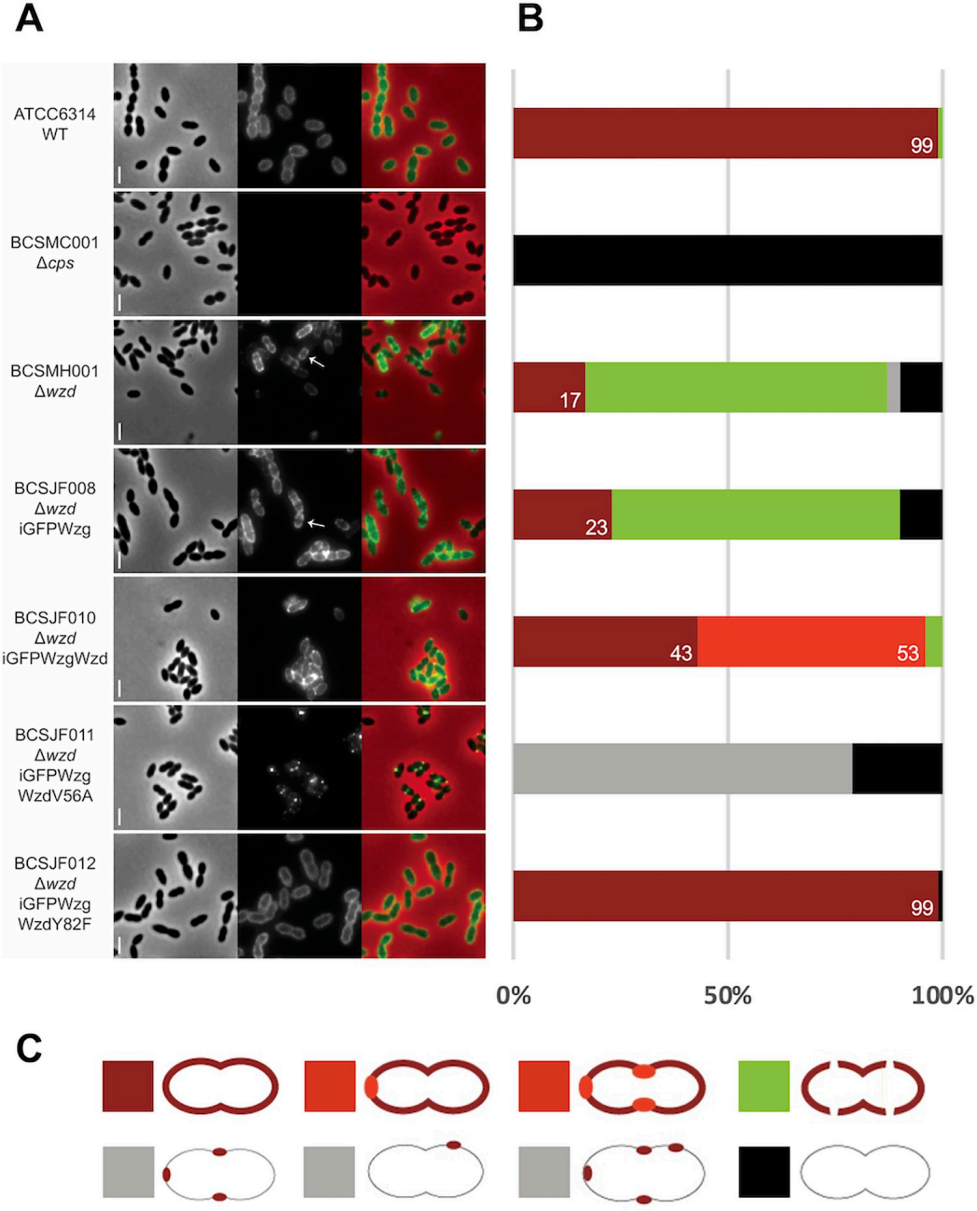
Expression of WzdV56A impairs the Wzg ability to produce a pneumococcal cell surface fully surrounded by capsule. A) Immunofluorescence microscopy images using a serotype-14 specific serum to detect the presence of the capsular polysaccharide at the cell surface. Wild-type encapsulated ATCC6314 (n = 115) expressed capsule all over the surface, while *wzd* null mutant strain BCSMH001 lacked capsule at midcell (n = 115). Cells of *wzd* null mutant strain were transformed with plasmids expressing (i) iGFP-Wzg alone (BCSJF008, n = 108), resulting in cells where the capsule is absent from the division septum; (ii) iGFP-Wzg and Wzd (BCSJF010, n = 104), or with WzdY82F (BCSJF012, n = 117), resulting in cells with homogeneous distribution of CPS at their surface or (iii) iGFP-Wzg and WzdV56A (strain BCSJF011, n = 105), resulting in cells where CPS accumulated in spots and was absent from most of the bacterial cell surface. Representative phase contrast (top panels, for visualization of bacteria), fluorescence microscopy (middle panels, for detection of the capsule associated with the bacterial cell surface) and overlay (bottom panels) images of each strain are shown. Arrows highlight bacteria that lack CPS at midcell. Scale bar, 2 μm. B) Graph shows the percentage of cells, for each strain, with different CPS patterns (grouped in 5 different classes). Numbers represent the percentage of cells fully covered with CPS. C) Classes of CPS patterns: cells fully covered with homogenous CPS (dark red); cells fully covered with CPS whose staining is heterogenous and has brighter regions (light red); cells partially covered with interruptions at the division septum (green); cells with CPS only in spots in particular regions (grey) or lacking CPS (black).

Given the proposed role of Wzg in the attachment of capsule polysaccharide to the peptidoglycan, we questioned if the WzdV56A mutant, which can localize at the division septum, but is unable to recruit the Wzg ligase, was affected in capsule distribution at the bacterial surface. In encapsulated pneumococcal cells, the capsule is distributed throughout the surface, while in a strain lacking Wzd, the capsule is detected at the cell surface, but it is absent from the division septum [28] (Fig 6). This is not due to an increased growth rate induced by the lack of capsule as the wild type ATCC6314 and its *wzd* null mutant (BCSMH001) strains have identical duplication times (33 min for both strains).

We transformed the *wzd* null mutant with plasmids encoding (i) iGFP-Wzg alone (strain BCSJF008); (ii) iGFP-Wzg and Wzd (strain BCSJF010); (iii) iGFP-Wzg and one of the variants WzdY82F (strain BCSJF012) or WzdV56A (strain BCSJF011) and visualized the presence of the capsule by immunofluorescence microscopy (Fig 6). Complementation of the *wzd* null mutant with a plasmid co-expressing iGFP-Wzg and Wzd, or WzdY82F, resulted in bacteria encapsulated, including at the septum. However, complementation with a plasmid co-expressing iGFP-Wzg with WzdV56A, resulted in bacteria that produce total capsule at similar levels as the parental strain but lower levels of CPS linked to the cell wall (S5 Fig). However, the CPS in cells expressing WzdV56A have a completely different pattern, as it was no longer homogeneously detected around the cells (Fig 6). Instead, CPS was absent from most regions of the cell and accumulated in dots near the division septum or at the cell poles. These CPS foci may reflect the subcellular localization of the machinery responsible for the polymerization and translocation of the CPS.

### Capsule absence at midcell results in increased exposure of bacterial septal cell wall

The coordination of the Wzd/Wze complex with the Wzg ligase may ensure that newly synthesized cell wall is simultaneously produced and masked by CPS, so that bacteria permanently present a fully concealed cell wall. Absence of CPS at the division septum, caused by the lack of Wzd, may result in exposure of particular cell surface components, such as PGN or wall teichoic acids, to receptors produced by the host immune system.

**Fig 7:**
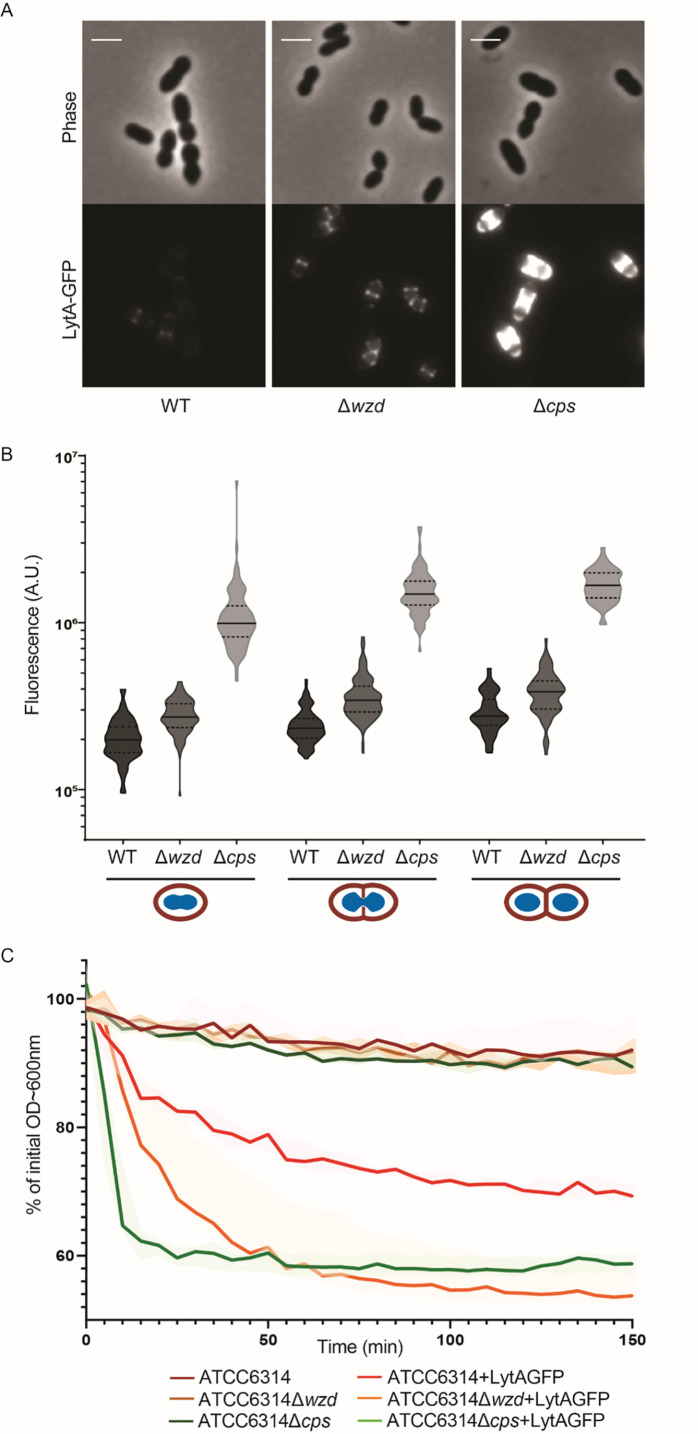
Absence of capsule at the division septum results in increased exposure of bacteria cell wall and susceptibility to lysis. A) Epifluorescence microscopy images of wild-type ATCC6314 strain (WT), its capsule null mutant (BCSMC001; Δ*cps*) and the *wzd* null mutant (BCSMH001; Δ*wzd*) incubated with LytA-GFP. Scale bar, 2 μm. B) Quantification of cell bound LytA-GFP fluorescence. Bacteria were grouped in three different classes (n> 50), depending on their cell cycle stage: (I) recently divided cells; (II) cells initiating division as seen from invagination of cell surface; (III) cells at the final steps of division, with deep invagination at division septum. Lack of capsule (at mid-cell or over the entire cell wall) results in bacteria that bind more LytA-GFP than the parental encapsulated bacteria. C) Lysis of boiled bacterial cells of wild-type ATCC6314, unencapsulated (ATCC6314Δ*cps*) and *wzd* null mutant (ATCC6314Δ*wzd*) strains was assessed by the decrease in OD 600 nm in the absence (n = 3) or presence (n = 7) of LytA-GFP. Lysis curves show that the lack of capsule at particular sub-cellular sites of the bacterial cell surface increase bacteria susceptibility to lysis. Solid lines represent the curve obtained with the median values for each timepoint. Shaded areas associated with each solid line represent the interquartile range.

To determine if wall teichoic acids are differently exposed at the bacterial cell surface in the absence of CPS, we used a recombinant and purified fluorescent derivative of LytA, a peptidoglycan hydrolase produced by *S*. *pneumoniae* that binds phophorylcholine residues present in pneumococcal wall teichoic acids [37]. We hypothesized that localized or complete absence of capsule, in the *wzd* or in the *cps* null mutants respectively, could lead to an increased number of LytA-GFP molecules bound to the surface of bacteria. Addition of LytA-GFP to growing cultures of the wild-type ATCC6314 strain, its *wzd* null mutant (BCSMH001) and the capsule null mutant (BCSMC001), followed by epifluorescence microscopy to quantify levels of bound protein, showed that the fluorescent signal of bound LytA-GFP was ~1.5 times higher in the *wzd* null mutant than in the parental encapsulated strain. As expected, this increase was more pronounced in the unencapsulated strain (BCSMC001), where the fluorescent signal from bound LytA-GFP was ~6 times higher than for the parental strain (Fig 7A and 7B). The observed increased levels of LytA-GFP were not a consequence of an increased cell volume, as *wzd* mutant cells were significantly smaller than parental and non-encapsulated bacteria (S6 Fig and S1 Table).

Increased exposure of the cell wall of *wzd* mutant to LytA-GFP could be deleterious for bacteria, as it could increase susceptibility to lysis by external PGN hydrolases. To test this hypothesis, purified LytA-GFP was added to previously boiled bacterial cultures (required to inactivate native PGN hydrolases). Both the *wzd* null mutant and *cps* null mutant were more susceptible to lysis than the parental encapsulated ATCC6314 strain (Fig 7C). Interestingly, the *wzd* null mutant, with partial exposure of the bacterial cell surface, was as susceptible to LytA-GFP induced lysis as *cps* null mutant, with complete absence of capsule. This suggests that even a small breach in the concealment provided by CPS to the bacterial cell wall is sufficient to expose bacteria to cell wall binding molecules present in the growth medium, such as PGN hydrolases or host immune receptors.

### Absence of capsule at midcell impairs virulence

To test the consequences of the absence of capsule at midcell on virulence, we used a zebrafish infection model. Due to its small size and rapid generation time, zebrafish (*Danio rerio*) is a powerful vertebrate model for studying host-pathogen interactions [38–41]. In particular, zebrafish embryos have been used as a model for host immune responses in systemic *S*. *pneumoniae* infections [42].

**Fig 8:**
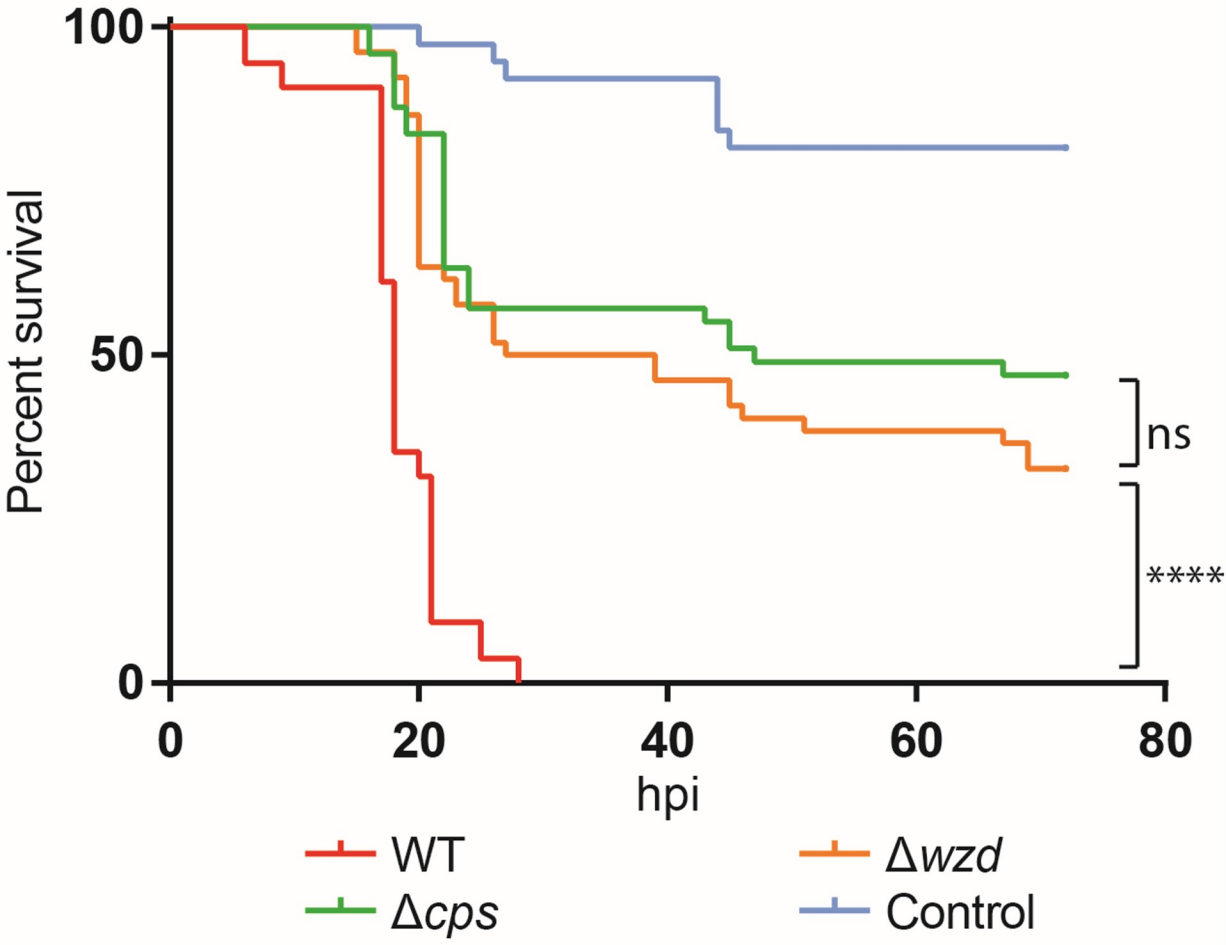
Full encapsulation of the cells is necessary for virulence. A) Survival graph of zebrafish embryos injected, three days post-fertilization, with wild-type ATCC6314 (WT), unencapsulated BCSMC001 (Δ*cps*), *wzd* null mutant BCSMH001 (Δ*wzd*) and medium used to resuspend bacteria (Control). The *wzd* null mutant was severely impaired in virulence, similarly to the unencapsulated BCSMC001 strain. The assay was performed with at least 50 embryos for each condition that were injected in four different days and the obtained survival curves, except those obtained with the *cps* and *wzd* mutants, were significantly different (p<0.0001). (hpi) hours post infection.

We performed survival assays by infecting zebrafish embryos, 3 days post-fertilization, with *S*. *pneumoniae* strains ATCC6314 (encapsulated), BCSMC001 (unencapsulated) and BCSMH001 (*wzd* null mutant). As observed in Fig 8, less than 30h post-infection, 0% of zebrafish embryos infected with the wild-type strain survived. However, 33% of embryos infected with the *wzd* null mutant strain (BCSMH001) were able to survive, even 72h after infection, close to the 47% of embryos that survive when infected with unencapsulated strain (BCSMC001). These results demonstrate that absence of capsule at the septum results in bacteria severely impaired in the ability to kill zebrafish embryos.

## Discussion

We have previously proposed that Wzd and Wze act as spatial regulators of capsule synthesis, to ensure that it occurs at the division septum, possibly to conceal the newly synthesized cell wall [28]. Therefore, Wzd and Wze guarantee that the *S*. *pneumoniae* cell wall is completely surrounded and protected by the capsular polysaccharide during the entire cell cycle. However, how Wzd and Wze perform this role was not known. Two hypotheses could be envisioned: Wzd/Wze could recruit other members of the CPS synthesis machinery to the division septum or, alternatively, Wzd/Wze could interact and activate other members of the CPS synthesis machinery already present at that site. To understand the role of Wzd/Wze, we used a BTH assay and tested whether WchA, the first glycosyltransferase in the synthesis of the CPS repeating unit, or Wzg, the CPS-cell wall ligase, interacted with Wzd or Wze. While no interactions were detected between Wzd/Wze and WchA, we found that Wzg interacts with Wzd, as well as with itself. LCP enzymes such as Wzg are generally thought to work as monomers [43], although LcpA from *Corynebacterium glutamicum* can dimerize under particular conditions [44], and that may also be the case in *S*. *pneumoniae*.

Importantly, this work showed that Wzd/Wzg interaction is critical for correct localization of Wzg. As expected for a protein with three transmembrane domains, Wzg was found to be present at the membrane of pneumococcal cells. A Wzg fluorescent derivative was enriched at the septum of wild-type encapsulated ATCC6314 (serotype 14) cells, as previously described for the *S*. *pneumoniae* encapsulated D39 (serotype 2) and unencapsulated R6 strains [18]. However, contrary to what was previously reported, we observed that the localization of Wzg was dependent on the presence of the *cps* operon, which is required for the ability of bacteria to produce capsule. Wzg lost its septal enrichment in a mutant strain lacking the *cps* operon and, specifically, in the absence of the Wzd or Wze proteins. It is possible that the role of Wzd on Wzg localization varies for different serotypes, explaining the discrepancies in data obtained by different groups.

Morona and colleagues have previously proposed that alterations in Wzd/Wze expression could influence the levels of capsule associated with the cell wall or released into the growth medium [19]. These authors found that the change of a valine to an alanine at position 56 of Wzd, or of a tyrosine to a phenylalanine at position 82, in bacteria that lack the Wzh phosphatase, causes a mucoid phenotype in pneumococcal serotype 2 colonies [19]. These mutants produce total CPS levels that are similar to the parental strain, but the levels of CPS actually attached to the cell wall are lower, suggesting a decreased activity of the CPS ligase. As we showed that Wzd and Wzg interact, leading to Wzg septal recruitment, we hypothesized that the V56A and Y82F mutations in Wzd might interfere with its interaction with and recruitment of Wzg. Indeed, WzdV56A did not interact with Wzg in a BTH assay and did not recruit Wzg to the division septum of pneumococcal cells. This was not the case for WzdY82F, which interacted with and recruited Wzg, indicating that the mucoid phenotype of this mutant arises via a different mechanism [19]. As substitution of Wzd tyrosine 82 by a cysteine was reported to cause a decrease in the phosphorylation of Wze [45], it is possible that this residue has a major role in the interaction between these two proteins or in the promotion of Wze phosphorylation by Wzd.

Wzg was initially thought to regulate transcription of the *cps* operon [16]. This was based on the homology of its C-terminal domain with LytR, a *B*. *subtilis* transcriptional regulator [15], and it was in accordance with a later report that showed that in *Streptococcus agalactiae*, the homologue protein CpsIA functions as a transcriptional regulator [46]. Moreover, Wzg homologues from *Streptococcus iniae* and *S*. *agalactiae* bind specifically to DNA containing the capsule operon promoter region [47,48]. It seems therefore plausible that Wzg has two roles in the regulation of the CPS assembly: (i) to attach the capsular polysaccharide to PGN, through its extracellular domain, although it has been reported that *wzg* insertion or deletion mutants can still attach CPS to the cell envelope [18,19,20], presumably through the activity of other LCP proteins; (ii) to control the transcription of the *cps* operon, through its short cytoplasmic N-terminal end. The fact that in a BTH assay the transmembrane domain and the first part of the extracellular domain of Wzg are required for an interaction with Wzd, and that a point mutation in the extracellular domain of Wzd prevents this interaction, highlights the importance of the extracellular regions of both proteins in *S*. *pneumoniae* CPS synthesis.

The role of Wzg, or of its successful interaction with Wzd, in the full encapsulation of the pneumococcal cell envelope may be specific to some serotypes, such as serotype 14, as it has been recently reported that CPS from serotypes 2, 8 and 31 is linked to peptidoglycan via a direct glycosidic bond, which would not require the role of this LCP protein [49], while serotype 14 CPS is connected through a phosphodiester bond to peptidoglycan which is labile to hydrofluoric acid [28]. We have further confirmed these results through sugar analysis of the cell envelope of serotype 14 cells. Hydrofluoric acid treatment released galactose, a sugar that is present in serotype 14 CPS, indicating that in these cells CPS is linked to peptidoglycan through a direct or indirect phosphodiester linkage.

More recently, the polymerase Wzy and the flippase Wzx were shown to localize exclusively at septum, suggesting that CPS synthesis occurs only at that place [29]. Interestingly, delocalization of Wzy was seen in a *wze* null mutant, which led to a sequential model where Wzd/Wze complex localize at the septum and then Wze captures Wzy resulting in its subsequent septal localization [29]. Considering these results, together with the data presented in this work, it is tempting to speculate that Wzd/Wze could control the elongation of the CPS chain (through recruitment of Wzy), as well as its ligation to the peptidoglycan (through recruitment of Wzg). Septal enrichment of Wzg may be crucial for CPS attachment to occur at a rate which is synchronized with the rate of PGN synthesis at the division septum. When this synchronization is lost, as it happens in the *wzd* mutant, where septal enrichment of Wzg is lost, *S*. *pneumoniae* cells synthesize PGN at the septum which is not immediately concealed by CPS. This results in cells where the cell wall at midcell is exposed to external WTA- or PGN-binding proteins. This model is line with the proposal that factor H binding proteins can protect the division septa of encapsulated bacteria against the host complement system [50]. In addition, in agreement with the idea that the septal cell surface requires a specific process of concealment, the *wzd* null mutant bound higher amounts of the PGN hydrolase LytA to its surface and was more susceptible to lysis by LytA than the fully encapsulated parental strain. More importantly, a *wzd* null mutant was dramatically impaired in virulence, in a zebrafish embryo infection model, a powerful vertebrate model for studying host-pathogen interactions due to its small size and rapid generation time [38–41], showing that full encapsulation of bacterial cells, covering the entire cell wall, is crucial for virulence. The observed small breaches in the concealment of bacteria, together with the reduced levels of CPS associated with bacteria, may explain why these mutants are unable to survive in the host.

## Materials and methods

### Ethics statement

Zebrafish larvae used in this study were 3 days post-fertilization and therefore are not subjected to any form of ethical regulation (European Union Directive 2010/63/EU). All the experiments were performed in strict compliance with national (DL 113/2013) and EU (Directive 2010/63/EU) regulations, in the i3S animal facility, with Association for Assessment and Accreditation of Laboratory Animal Care International (AAALAC) accreditation, that ensures high-quality care, providing animals with environmental enrichment, regular cleaning and daily checks. All personnel involved in animal handling and experimentation have individual training and authorization as per National/EU regulations.

### Bacterial strains and growth conditions

Bacterial strains and plasmids used in this study are listed in Table 1.

*Escherichia coli* bacteria were routinely grown in Luria Broth (LB) medium at 37°C, unless otherwise indicated. When appropriate, ampicillin (100 μg/ml) or kanamycin (50 μg/ml) were added to the growth media. For bacterial two-hybrid assays, MacConkey agar medium (Difco) and Maltose 1% (w/v) (Difco) were used.

**Table 1:** Bacterial strains and plasmids.

Name	Relevant characteristics	Source/Reference
Strains		
*E*. *coli*		
DH5α	*recA endA1 gyrA96 thi-1 hsdR17 supE44 relA1* ɸ80 d*lac*Z M15	Gibco-BRL
BL21(DE3)	*F–ompT gal dcm lon hsdSB(rB-mB-) λ(DE3 [lacI lacUV5-T7 gene 1 ind1 sam7 nin5])*	Lab Stock
BTH101	Reporter strain for BTH system, F^-^ *cya*-99, *ara*D139, *gal*E15, *gal*K16, *rps*L1 (Str^r^), *hsd*R2, *mcr*A1, *mcr*B1	[31]
BCSRN001	DH5α transformed with pBCSRN001 (pET21aLytA-GFP)	This work
*S*. *pneumoniae*		
ATCC6314	Encapsulated strain, serotype 14	American Type Culture Collection.
R36A	Non-encapsulated laboratory strain	[63]
BCSMC001	ATCC6314Δ*cps*	[28]
BCSMH001	ATCC6314Δ*wzd*	[28]
BCSMH002	ATCC6314Δ*wze*	[28]
BCSMH022	BCSMH001 transformed with pBCSMH007 (*wzd*-*citrine*)	[28]
BCSMH036	R36A transformed with pBCSMH020 (*sfGFP*)	[28]
BCSMH061	BCSMH001 transformed with pBCSMH064 (*wzdY39C-citrine*)	This work.
BCSMH062	BCSMH001 transformed with pBCSMH065 (*wzdV56A-citrine*)	This work.
BCSMH064	BCSMH001 transformed with pBCSMH066 (*wzdY82F-citrine*)	This work.
BCSMH065	BCSMH001 transformed with pBCSMH067 (*wzdV116A-citrine*)	This work.
BCSMH070	ATCC6314 transformed with pBCSMH070 (*wchA-CFP*)	This work.
BCSMH071	ATCC6314Δ*cps* transformed with pBCSMH070 (*wchA-CFP*)	This work.
BCSMH072	ATCC6314 transformed with pBCSMH071 (*iCFP-wzg*)	This work.
BCSMH073	ATCC6314Δ*cps* transformed with pBCSMH071 (*iCFP-wzg*)	This work.
BCSMH074	ATCC6314Δ*wze* transformed with pBCSMH071 (*iCFP-wzg*)	This work.
BCSMH075	ATCC6314Δ*wzd* transformed with pBCSMH071 (*iCFP-wzg*)	This work.
BCSMH076	ATCC6314Δ*cps* transformed with pBCSMH072 (*iCFP-wzgwzd*)	This work.
BCSMH077	ATCC6314Δ*wzd* transformed with pBCSMH072 (*iCFP-wzgwzd*)	This work.
BCSJF005	ATCC6314 transformed with pBCSJF005 (*iGFP-wzg*)	This work.
BCSJF006	BCSMC001 transformed with pBCSJF005 (*iGFP-wzg*)	This work.
BCSJF007	BCSMH002 transformed with pBCSJF005 (*iGFP-wzg*)	This work.
BCSJF008	BCSMH001 transformed with pBCSJF005 (*iGFP-wzg*)	This work.
BCSJF009	BCSMC001 transformed with pBCSJF006 (*iGFP-wzgwzd*)	This work.
BCSJF010	BCSMH001 transformed with pBCSJF006 (*iGFP-wzgwzd*)	This work.
BCSJF011	BCSMH001 transformed with pBCSJF007 (*iGFP-wzgwzdV56A*)	This work.
BCSJF012	BCSMH001 transformed with pBCSJF008 (*iGFP-wzgwzdY82F*)	This work.
BCSSP001	ATCC6314Δ*wzg*	This work.
BCSSP002	BCSSP001 transformed with pBCSMH073 (*wzg-GFP*)	This work.
BCSSP003	ATCC6314Δ*wchA*	This work.
BCSSP004	BCSSP003 transformed with pBCSMH070 (*wchA-CFP*)	This work.
BCSJC048	BCSMH036 transformed with pBCSJC044 (*lytA-GFP*)	This work.
Plasmids		
Plasmids for protein purification		
pET21a	T7p, T7t, His-Tag, lacI, Amp^r^	Lab Stock
pBCSRN001	pET21a containing *lytA-GFP*, Amp^r^	This work.
Plasmids for Bacterial Two-Hybrid Assay		
pUT18C	BTH plasmid, N-terminal *cyaA*T18 fusion, Amp^r^.	[31]
pUT18	BTH plasmid, C-terminal *cyaA*T18 fusion, Amp^r^.	[31]
pKT25	BTH plasmid, N-terminal *cyaA*T25 fusion, Kan^r^.	[31]
pKNT25	BTH plasmid, C-terminal *cyaA*T25 fusion, Kan^r^.	[31]
pKT25zip	BTH control plasmid, Kan^r^.	[31]
pUT18Czip	BTH control plasmid, Amp^r^.	[31]
pBCSMC001	BTH plasmid containing *cyaA*T25-*wzd* fusion.	[28]
pBCSMC002	BTH plasmid containing *cyaA*T18-*wze* fusion.	[28]
pBCSMC005	BTH plasmid containing *cyaA*T18-*wzd* fusion.	This work.
pBCSMC006	BTH plasmid containing *wze- cyaA*T18 fusion.	This work.
pBCSMC007	BTH plasmid containing *wzd- cyaA*T25 fusion.	This work.
pBCSMC008	BTH plasmid containing *cyaA*T25-*wze* fusion.	This work.
pBCSMC009	BTH plasmid containing *wze- cyaA*T25 fusion.	This work.
pBCSMH040	BTH plasmid containing *wzd- cyaA*T18 fusion.	This work.
pBCSMH049	BTH plasmid containing *cyaA*T18-*wzg* fusion.	This work.
pBCSMH050	BTH plasmid containing *wzg- cyaA*T18 fusion.	This work.
pBCSMH051	BTH plasmid containing *cyaA*T25-*wzg* fusion.	This work.
pBCSMH052	BTH plasmid containing *wzg- cyaA*T25 fusion.	This work.
pBCSMH053	BTH plasmid containing *cyaA*T18-*wchA*fusion.	This work.
pBCSMH054	BTH plasmid containing *wchA- cyaA*T18 fusion.	This work.
pBCSMH062	BTH plasmid containing *wzdV56A- cyaA*T18 fusion.	This work.
pBCSMH063	BTH plasmid containing *wzdY82F- cyaA*T18 fusion.	This work.
pBCSJF009	BTH plasmid containing *cyaA*T25-*wchA* fusion.	This work.
pBCSJF010	BTH plasmid containing *wchA*-*cyaA*T25 fusion.	This work.
Replicative plasmids in *S*. *pneumoniae*		
pBCSLF001	High-copy-number vector, contains the -10 constitutive promoter of *SigA* from *S*. *pneumoniae*, Tet^r^	[28]
pBCSMH002	Allows expression of Citrine fusion proteins, Tet^r^.	[28]
pBCSMH007	pBCSMH002 containing *wzd-citrine*, Tet^r^.	[28]
pBCSMH018	Allows expression of CFP fusion proteins, Tet^r^.	[28]
pBCSMH020	pBCSLF001 derivative, allows expression of sfGFP fusion proteins, Tet^r^.	[33]
pBCSMH031	Allows expression of CFP containing the first 10 aa of Wze at its N-terminus, Tet^r^.	[33]
pBCSMH032	Expression of sfGFP containing the first 10 aa of Wze at its N-terminus, Tet^r^.	[33]
pBCSMH064	pBCSMH002 containing *wzdY39C-citrine*, Tet^r^.	This work.
pBCSMH065	pBCSMH002 containing *wzdV56A-citrine*, Tet^r^.	This work.
pBCSMH066	pBCSMH002 containing *wzdY82F-citrine*, Tet^r^.	This work.
pBCSMH067	pBCSMH002 containing *wzdV116A-citrine*, Tet^r^.	This work.
pBCSMH070	pBCSMH018 containing *wchA-CFP*, Tet^r^.	This work.
pBCSMH071	pBCSMH031 containing *iCFP-wzg*, Tet^r^.	This work.
pBCSMH072	pBCSMH031 containing *iCFP-wzgwzd*, Tet^r^.	This work.
pBCSMH073	pBCSMH020 containing *wzg-GFP*, Tet^r^.	This work.
pBCSJF005	pBCSMH032 containing *iGFP-wzg*, Tet^r^.	This work.
pBCSJF006	pBCSMH032 containing *iGFP-wzgwzd*, Tet^r^.	This work.
pBCSJF007	pBCSMH032 containing *iGFP-wzgwzdV56A*, Tet^r^.	This work.
pBCSJF008	pBCSMH032 containing *iGFP-wzgwzdY82F*, Tet^r^.	This work.
pBCSJC044	pBCSMH020 containing *lytA-GFP*, Tet^r^.	This work.

*Streptococcus pneumoniae* bacteria were grown in liquid semi-defined C + Y medium [51] at 37°C, without aeration, or on tryptic soy agar (TSA, Difco) plates supplemented with 5% (v/v) sheep blood (ThermoScientific). When needed, tetracycline (1 μg/ml) was added to the growth media.

### DNA manipulation procedures

*E*. *coli* and *S*. *pneumoniae* competent cells preparation and transformation were performed as previously described [52,53]. PCR products and plasmid DNA were purified with Wizard SV Gel and PCR Clean-up System and Wizard Plus SV Minipreps, respectively (Promega). PCR fragments were amplified with Phusion High-Fidelity DNA polymerase (Finnzymes). Restriction enzymes used were acquired from New England Biolabs or from Fermentas. Primers used in this work are listed in Table 2.

**Table 2:** Primers used in this work.

Primers	Sequence 5’➔3’	Features/ Restriction sites
1	CGGGATCCCGGAGAAAATATGAAGG	BamHI
2	GGAATTCCCCTCTCTCCTATTTC	EcoRI
3	CTAGTCTAGAGATGAAGGAACAAAACACTTTGG	XbaI
4	CGCGGATCCCCTTTCAACTTACTCAAGTTTGG	BamHI
5	CGGGATCCTAAGGAGGAAATATGA	BamHI
6	GGAATTCCATTTCAACTTACTCAAG	EcoRI
7	CGGGATCCTAGGAGGGAGGAATGCCG	BamHI
8	GGAATTCCATTTTTTACCATAATTTCC	EcoRI
9	CGGGATCCAATGCCGACATTAGA	BamHI
10	GGAATTCCCCTAAGTTATTTTTTACC	EcoRI
11	CGCGGATCCAATGAGTAGACGTTTTAAAAAATCACG	BamHI
12	CCGGAATTCTCATCTACCCTCCATCACATCC	EcoRI
13	CCGGAATTCTTTCTACCCTCCATCACATCC	EcoRI
14	AACTGCAGAATGGATAAAAAAGGATTGG	PstI
15	CGCGGATCCTTACTTCGCTCCATTTCTC	BamHI
16	CGCGGATCCTTCTTCGCTCCATTTCTC	BamHI
17	GGCTGCAGAGATGGATAAAAAAGGATTGG	PstI
18	CGCGGATCCTTACTTCGCTCCATTTCTC	BamHI
19	AACTGCAGAATGGATAAAAAAGGATTGG	PstI
20	CGCGGATCCTTCTTCGCTCCATTTCTC	BamHI
21	CTAGCTAGCATGAAGGAACAAAACACTTTGG	NheI
22	GTACT**GCA**GGCAAAAGC	Mutation Y➔C
23	GCTTTTGCC**TGC**AGTAC	Mutation Y➔C
24	GGGGTACCTTTCAACTTACTCAAG	KpnI
25	CGGTTAAC**TGC**ATAAATCCG	Mutation V➔A
26	CGGATTTAT**GCA**GTTAACCG	Mutation V➔A
27	GATAATTTCACG**AAA**GTCTTTAAC	Mutation Y➔F
28	GTTAAAGAC**TTT**CGTGAAATTATC	Mutation Y➔F
29	GTATCAACTGG**TGC**TGTTAC	Mutation V➔A
30	GTAACA**GCA**CCAGTTGATAC	Mutation V➔A
31	ATAAGAATGCGGCCGCAATGAGTAGACGTTTTAAAAAATCACG	NotI
32	GAAGATCTTCATCTACCCTCCATCACATCC	BglII
33	GGAAGATCT**TAAGGAGGT**GCTGCGGCCATGAAGGAACAAAACAC	BglII; **RBS**
34	GAAGGCCTCTATTTCAACTTACTCAAG	StuI
35	CTAGCTAGCATGGATAAAAAAGGATTGG	NheI
36	CGCGCCGGCCTTCGCTCCATTTCTCATAAATAC	NaeI
37	GGCGCTAGCATGGAAATTAATGTGAGTAAATTAAG	NheI
38	GCGGTACCTTTTACTGTAATCAAGCCATC	KpnI
39	GCGGGATCCATGGAAATTAATGTGAGTAAATTAAG	BanHI
40	GCGCTCGAGGTCGACTTTGTATAGTTCATC	XhoI
41	GTACCCCTATGCAATGGGACG	Forward primer for upstream region of *wzg*
42	CTACTATCATCGATTAACACCTATACCTTGAACATCGTAC	Reverse primer for upstream region of *wzg* with overlap with downstream
43	GGTGTTAATCGATGATAGTAGTTTAGCTGTAGTTAAAGC	Forward primer for downstream region of *wzg* with overlap with upstream
44	GCTATTTCTAATGTCGGCATTCC	Reverse primer for downstream region of *wzg*
45	GGAACGTGATTTAGTTCATGTAG	Forward primer for upstream region of *wchA*
46	CTTTCCAGATTGTCGTTATTTTTTACCATAATTTCC	Reverse primer for upstream region of *wchA* with overlap with downstream
47	GGTAAAAAATAACGACAATCTGGAAAGATATTG	Forward primer for downstream region of *wchA* with overlap with upstream
48	CCGTCCCAGTCTAACAAAC	Reverse primer for downstream region of *wchA*

### Construction of plasmids for Bacterial Two-Hybrid assays

Plasmids to test Wzd and Wze interactions with other capsule proteins, were constructed using inserts amplified from ATCC6314 chromosomal DNA, unless otherwise indicated.

Plasmids pBCSMC005, pBCSMH040 and pBCSMC007, encoding fusion proteins T18-Wzd, Wzd-T18 and Wzd-T25, were constructed by amplification of *wzd* with primers 1/2, 3/4 and 5/6, respectively, followed by restriction and ligation into plasmids pUT18C, pUT18 and pKNT25.

Plasmids pBCSMC006 and pBCSMC009, encoding fusions Wze-T18 and Wze-T25 were constructed by amplification of *wze* with primers 7/8, followed by restriction and ligation into pUT18 and pKNT25, respectively. Plasmid pBCSMC008, encoding fusion T25-Wze, was obtained by amplification of *wze* with primers 9/10 and cloning into pKT25.

Plasmids pBCSMH049 and pBCSMH051, encoding T18-Wzg and T25-Wzg, were constructed by amplification of *wzg* with primers 11/12, restriction and ligation into pUT18C and pKT25. Amplification of *wzg* with primers 11/13, restriction and ligation into pUT18 and pKNT25 produced plasmids pBCSMH050 and pBCSMH052, encoding the fusions Wzg-T18 and Wzg-T25, respectively.

Plasmids pBCSMH053 and pBCSMH054, encoding T18-WchA and WchA-T18, were constructed by amplification of *wchA* with primer pairs 14/15 and 14/16, followed by restriction and ligation into pUT18C and pUT18, respectively.

Plasmids pBCSJF009 and pBCSJF010, encoding fusions T25-WchA and WchA-T25 were constructed by amplification of *wchA* with primer pairs 17/18 and 19/20, respectively, restriction and ligation into pKT25 and pKNT25.

Plasmids pBCSMH062 and pBCSMH063, encoding fusions WzdV56A-T18 and WzdY82F-T18, were constructed using primers 3/4 to amplify the mutated *wzd* genes from plasmids pBCSMH064 and pBCSMH066, respectively, followed by restriction and ligation into pUT18.

The nucleotide sequences of the inserts of the constructed plasmids were confirmed by sequencing.

### Bacterial two-hybrid assays

Bacterial Two-hybrid assays were done in *E*. *coli* strain BTH101 [31]. Transformants with plasmids mentioned above were plated in MacConkey media, supplemented with ampicillin, kanamycin and maltose. Plates were incubated at 30°C and screened for pink/white colonies, in which pink indicated a positive interaction. Single colonies were grown at 30°C in the presence of 0.5 mM of IPTG and the interactions confirmed by β-galactosidase activity measurements as described [54]. Part of these cultures (3 μl) were also used to spot fresh plates to visualize differences in the color of the bacterial lawn.

### Construction of plasmids for protein expression in *S*. *pneumoniae*

To determine the localization of Wzd proteins containing the point mutations Y39C, V56A, Y82F and V116A, plasmids expressing Citrine C-terminal fusions of the mutated proteins were constructed. The mutated *wzd* genes were amplified in two different fragments using primers 21/22 and 23/24 for *wzdY39C*; 21/25 and 26/24 for *wzdV56A*; 21/27 and 28/24 for *wzdY82F*; 21/29 and 30/24 for *wzdV116A*. In each case, the two fragments were joined by overlap extension PCR using primers 21/24 and cloned in pBCSMH002, producing plasmids pBCSMH064-067.

Plasmid pBCSJF005, encoding a fluorescent derivative of Wzg, was constructed by amplification of *wzg* from the ATCC6314 chromosomal DNA with primers 31/32, restriction and ligation into pBCSMH032.

Plasmids pBCSJF006-008 were constructed by amplification of *wzd*, *wzdV56A* and *wzdY82F*, respectively, with primers 33/34 using plasmids pBCSMH007, pBCSMH065 and pBCSMH066 as templates, restriction and ligation into pBCSJF005. In the resulting plasmids, the protein fusion iGFP-Wzg is expressed in the presence of Wzd, WzdV56A or WzdY82F.

CFP fluorescent derivatives of Wzg were expressed through the construction of plasmid pBCSMH071 by amplification of *wzg* from the ATCC6314 chromosomal DNA with primers 31/32, restriction and ligation into pBCSMH031. Amplification of *wzd* with primers 33/34 using plasmid pBCSMH007 as template, restriction, and ligation into pBCSMH071 produced plasmids pBCSMH072. In this plasmid, the protein fusion iCFP-Wzg is expressed in the presence of Wzd.

Plasmid pBCSMH070, was constructed by amplification of *wchA* from the ATCC6314 chromosomal DNA with primers 35/36, restriction and ligation into pBCSMH018.

Plasmid pBCSJC044, encoding a LytA fluorescent derivatives, was constructed by amplification of *lytA* with primers 37/38, restriction and ligation into pBCSMH020.

The nucleotide sequences of the inserts of the constructed plasmids were confirmed by sequencing.

### Construction of plasmids for protein expression in *E*. *coli*

To express in *E*. *coli* and purify the protein LytA-GFP, amplification of *lytAGFP* was done using primers 39 and 40 and the plasmid pBCSJC044 as template. Restriction and ligation into pET21a, produced plasmid pBCSRN001.

### Construction of null mutants of *S. pneumoniae*

Strains ATCC6314Δ*wzg* and ATCC6314Δ*wchA* were constructed using the method described by Dalia and colleagues [55]. The upstream and downstream regions of target genes were amplified by overlap extension PCR to produce a single fragment. The *rpsL1* gene cassette carrying streptomycin resistance was used for co-transformation experiments, using the corresponding overlap extension PCR amplicon, into the target *S*. *pneumoniae* strain. Selection of transformants was made in the presence of streptomycin (100 μg/mL). For the construction of the ATCC6314Δ*wzg* null mutants we used the primers 41–44. For the construction of the ATCC6314Δ*wchA* null mutants we used the primers 45–48.

### Protein purification

The protocol for the purification of *S*. *pneumoniae* LytA-GFP was adapted from [56]. Briefly, *E*. *coli* BL21(DE3) cells were transformed with the plasmid pBCSRN001 and incubated overnight at 37°C, with vigorous shaking, in LB supplemented with 100 μg/ml ampicillin and 2% (m/v) lactose. Cells were harvested by centrifugation, resuspended in 20 mM sodium phosphate buffer, pH 6.9, and broken by sonication. Clarified lysate was applied to DEAE-cellulose resin (DEAE Sephacel, GE Healthcare) and incubated at 4°C for 1 h with stirring. Bound protein was washed five times with 20 mM sodium phosphate buffer containing 1.5 M NaCl, then eluted in the same buffer containing 2% (m/v) choline. Protein was dialyzed against 20 mM sodium phosphate buffer, pH 6.9 to remove the salts and aliquots were stored at 4°C.

### Lysis assay

For lysis assays, encapsulated ATCC6314, its capsule null mutant (BCSMC001) and the *wzd* null mutant (BCSMH001) strains were grown O/N in 50ml of C+Y until OD~0.9, centrifuged, washed once with fresh C+Y medium, resuspended in 50 ml, and placed for 20 minutes in an ice bath. Cell suspensions were added to boiling water, at a flow that did not stop boiling, and further boiled for 40 minutes. Cell suspensions were then diluted to OD~1 and divided in two tubes, one of which received purified LytA-GFP at a final concentration of 6,75 μg/ml. OD600nm was followed in a Cary 100 UV-Vis Spectrophotometer (Agilent) using 10x10 mm cuvettes (Sarstedt) containing 3ml of culture, with a sterile gauze lid and a cuvette-adapted stirring bar (VWR) to allow continuous slow stirring. Lysis was followed by the decrease in OD600nm measured every 5 minutes for 150 minutes.

### Growth assays

*S*. *pneumoniae* cells were grown O/N at 37°C without aeration in liquid semi-defined C + Y medium. When stationary phase was reached, at an OD~0.8, cultures were diluted to OD~0.05. The OD600nm was measured every 30 minutes until OD~0.2 and then every 15 minutes. Absorbance values obtained during exponential growth were selected and used to determine duplication times (presented as a median value and n = 10).

### Microscopy

*S*. *pneumoniae* strains were grown until early exponential phase and observed by fluorescence microscopy on a thin layer of 1% (m/v) agarose in PreC medium [51]. Images were acquired in a Zeiss Axio Observer microscope, equipped with a Photometrics CoolSNAP HQ2 camera (Roper Scientific), with appropriate exposure times (500–1000 ms for GFP; 5000 ms for Citrine and CFP; 100 ms for AlexaFluor secondary antibody), and analyzed using FIJI software [57] as well as eHooke software [58], which was developed in-house and is available at https://github.com/BacterialCellBiologyLab/eHooke.

Determination of the fluorescence ratio (FR) was performed as previously described [35]. Briefly, the intensity of the fluorescent signal at the division septum was divided by the fluorescent signal at the peripheral membrane. Average background fluorescence was subtracted from every value. An FR value higher than 2 is indicative of septal enrichment. Quantification was performed for at least 100 cells of each strain.

In vivo detection of the capsule produced at the surface of *S*. *pneumoniae* cells was performed as previously described [28], but Anti-rabbit Alexa Fluor 594 antibody (Invitrogen) was used as a secondary antibody. When necessary LytA-GFP purified protein was added to the media at 5 μg/ml concentration and incubated for 10 min at 37°C.

### Determination of the CPS associated with pneumococcal cells

Cells were harvested at exponential growth-phase (OD_(600nm)_ ~ 0.5) by centrifugation, washed with one volume of fresh C+Y medium and resuspended in water. After adjusting samples to same cell density, cells were lysed with deoxycholate (0.25 mg/ml for 30 min at 37°C) and boiled for 3 min before use. Samples of purified cell walls were prepared as previously described [59]. Briefly, cells were boiled into sodium dodecyl sulfate (SDS, final concentration, 4% (m/v)) for 30 min to inactivate any enzyme that could modify the bacteria cell wall. After removal of SDS, cell walls were mechanically broken by shaking with an equal volume of acid-washed glass beads with a FastPrepFP120 apparatus. Cell walls were digested with Dnase and Rnase (for 3 h at 37°C), and trypsin (overnight at 37°C), which were inactivated by boiling in 1% (m/v) (final concentration) SDS. Cell walls were washed twice with water, once with 8 M LiCl and then with 100 mM EDTA. Before lyophilization, broken cell walls (CW) were washed three times with water. In order to load in the dot-blot similar amounts of cell walls, the content of muramic acid was determined in each purified sample using HPAEC-PAD (High Performance Anion Exchange Chromatography coupled with Pulsed Amperometric Detection at the Chemical Analysis Laboratory at REQUIMTE-LAQV as previously described [60]. Samples were loaded onto Nitrocellulose (Amersham) membranes, which had been pre-equilibrated in PBS and placed on top of PBS soaked Hybond Blotting Paper. A volume of 3ul of each sample was loaded in triplicates, membranes were allowed to air-dry for 30 min and then blocked during 1 h in Blocking Buffer (5% (m/v) non-fat dried milk in PBS). Membranes were washed 3 times in PBS-T (PBS + 0.05% (v/v) Tween 20) and incubated overnight at 4°C with primary Anti-CPS14 antibody diluted 3/1000 in PBS-T, purified as previously described [28,61]. After washing with PBS-T, membranes were incubated during 1 h at room temperature with the secondary antibody Anti-Rabbit StarBright Blue 700 (BioRad) diluted 1/5000 in PBS-T. Membranes were again washed 3 times with PBS-T and detected using the iBright FL1500 Imaging System.

Images were analyzed using FIJI software [57] to determine the average intensity present in each dot-blot spot. Average background fluorescence was subtracted from every value. Quantification was performed in membranes prepared in three independent days, each with 3–6 replicate spots from each sample.

### Infection assays using zebrafish embryos

Wild-type AB zebrafish were obtained from the Zebrafish International Resource Center (Eugene). Embryos were raised at 28°C in E3 medium. Larvae were anaesthetized with 200 μg/ml tricaine (Sigma) for the injection procedure. For injection of zebrafish larvae, cells were grown at 37°C without aeration in liquid semi-defined C + Y medium, harvested (1 ml) at exponential growth (OD600nm~0.6), washed with fresh C+Y medium and resuspended in 100 μl of C+Y medium. A 70 kDa rhodamine dextran (Invitrogen/Molecular Probes) tracer was added in a proportion of 1:1 just before injection. Anaesthetized zebrafish larvae, 3 days post-fertilization (dpf), were microinjected in the hindbrain ventricle with 0.5–2 nl of bacterial suspension [62], so that a group of 12 embryos for each bacterial strain were injected with the same glass microcapillary needle filled with bacterial suspension. This experiment was repeated four times. Bacterial load upon infection was determined by plating at least three alive larvae onto TSA 5% (v/v) blood agar before and 2 hours post-injection, which confirmed that the number of bacteria injected for each strain were very similar. Each larvae was collected into an Eppendorf with 200 μl of lysis solution and then were mechanically smashed dilutions in C+Y were plated. Infected larvae were transferred into individual wells (containing 1 ml of E3 in 24 well plates), incubated at 30°C and regularly observed under a stereomicroscope [42].

### Statistical analysis

Statistical analyses of data presented in the figures were done using GraphPad Prism 8 software (GraphPad Software).

Analysis of data of the beta-galactosidase activity presented in Figs 1, 3, 5 and S2; of the Fluorescence ratios (FR) presented in Figs 2, 5, 7 and S3 and S4; and of the Area of bacteria in S6 Fig was done using a Kruskal-Wallis Test, and a Mann-Whitney Test. Results are available in S1 Table. P values ≤ 0.05 were considered as significant for all analysis performed and are indicated with asterisks: *P ≤ 0.05, **P ≤ 0.01, ***P ≤ 0.001 and ****P ≤ 0.0001.

Survival rates of the zebrafish embryos in the experiments reported in Fig 8, were also analyzed with the Graph Pad Prism 8 software. The statistical significance in these assays was measured using the Log-rank (Mantel-Cox) test. P values of <0.05 were considered significant.

## Supporting information

S1 Fig Schematic representation of the *cps* operon of serotype 14 ATCC6314 strain. Represented in figure are serotype specific genes that encode several glycosyltransferases that link or modify the different sugars present in the capsule repeating unit (light blue); that attach the first sugar of the repeating unit to the lipid anchor (WchA, light orange); the *wzx* gene (purple), which encodes the transporter of the CPS repeating unit from the inner to the outer face of the bacterial membrane; the *wzy* gene (orange), which encodes the polymerase involved in the polymerization of different repeating units and in the assembly of a mature CPS. The first four genes at the 5’ end of the *cps* operon are highly conserved between serotypes and are proposed to be involved in the regulation of the synthesis of CPS. The first gene is *wzg* (dark blue), which encodes a ligase capable of attaching the capsule to the peptidoglycan macromolecule. The other three regulatory genes (yellow) encode Wze, an autophosphorylating tyrosine kinase; Wzd, a membrane protein required for the autophosphorylation of Wze and Wzh, a phosphotyrosine protein phosphatase that dephosphorylates Wze. Data adapted from Bentley et al. (14).
(TIF)

S2 Fig Introduction of the T25 tag at the C-terminal of different capsule synthesis proteins may prevent their interaction with partner proteins. Wze, Wzd, Wzg and WchA interactions were tested using Bacterial Two-Hybrid *E*. *coli* system. β-galactosidase activity of cells expressing putative interaction partners was measured in cell extracts in at least three independent replicates. Black circles indicate median values and brackets show the 25% and 75% percentiles. Positive control (+): *E*. *coli* expressing T18 and T25 fragments linked to leucine zipper domains (zip) that can dimerize; Negative control (-): *E*. *coli* expressing untagged T18 and T25 fragments. No interactions were detected between tested proteins.
(TIF)

S3 Fig **A) Expression of iGFPWzg complements the ability of ATCC6314 *wzg* null to produce capsule.** Immunofluorescence microscopy images using a serotype-14 specific serum to detect the presence of the capsular polysaccharide at the cell surface show that all cells of wild-type encapsulated ATCC6314 strain are surrounded by the capsule over the entire surface and that this number is reduced to 45% in its *wzg* null mutant strain (BCSSP001). Expression of iGFPWzg encoded in a replicative plasmid in BCSSP002 strain allows the expression of capsule in 95% of bacteria. Representative phase contrast (top panels, for visualization of bacteria) and fluorescence microscopy (middle panels, for detection of the capsule associated with the bacterial cell surface) images of each strain are shown. Scale bar, 2 μm. B) **Septal localization of Wzg is dependent on the expression of Wzd/Wze.** Graph shows the ratio of iCFP-Wzg fluorescence measured at the septum versus the peripheral wall in the *S*. *pneumoniae* wild-type encapsulated strain (BCSMH072, n = 132), the capsule null mutant (BCSMH073, n = 129), the *wze* null mutant (BCSMH074, n = 130), the *wzd* null mutant (BCSMH075, n = 112) and in the *cps* and *wzd* null mutants expressing Wzd from a constitutive promoter (BCSMH076, n = 135, and BCSMH077, n = 154, respectively). Enrichment of Wzg at the septum is only observed when Wzd is expressed and is localized at the division septum. Solid lines indicate median, and dashed lines indicate 25% and 75% percentiles. Representative phase contrast and fluorescence microscopy images of each strain are shown below the graph. Scale bar, 2 μm.
(TIF)

S4 Fig **A) Membrane localization of WchA is independent of the presence of the *cps* operon.** Graph shows the ratio of WchA-CFP fluorescence measured at the septum versus the peripheral wall in the *S*. *pneumoniae* wild-type encapsulated strain (BCSMH070, n = 101) and in the capsule null mutant (BCSMH071, n = 101). No difference in the localization of WchA was observed between encapsulated and non-encapsulated bacteria. Solid lines indicate median, and dashed lines indicate 25% and 75% percentiles. Representative phase contrast and fluorescence microscopy images of each strain are shown below the graph. Scale bar, 2 μm. **B) Expression of WchACFP complements the ability of ATCC6314 *wchA* null to produce capsule.** Immunofluorescence microscopy images using a serotype-14 specific serum to detect the presence of the capsular polysaccharide at the cell surface show that all cells of wild-type encapsulated ATCC6314 strain are surrounded by the capsule over the entire surface and that no cells expressing capsule can be observed in its *wchA* null mutant strain (BCSSP003). Expression of WchACFP encoded in a replicative plasmid in BCSSP004 strain allows the expression of capsule in most bacteria. Representative phase contrast (top panels, for visualization of bacteria) and fluorescence microscopy (middle panels, for detection of the capsule associated with the bacterial cell surface) images of each strain are shown. Scale bar, 2 μm.
(TIF)

S5 Fig Expression of capsular polysaccharide in the presence of different Wzd proteins. **A)** Cells from exponentially growing cultures of ATCC6314 (encapsulated parental strain, WT), BCSMC001 (non-encapsulated Δ*cps* mutant strain), BCSMH001 (Δ*wzd* mutant strain) and its derivatives strains that carry a plasmid expressing iGFPWzg in the presence of Wzd (BCSJF010 strain); of the mutated WzdV56A protein (BCSJF011 strain) and of the mutated WzdY82F protein (BCSJF012 strain) were analysed by dotblot using serotype-14 specific serum. Graph shows the intensity of the fluorescence signal measured in dot-blot assays in three independent experiments. Expression of WzdY82F and WzdV56A in the *wzd* null mutant strain allows expression of capsule at levels similar to those observed with the parental strain. **B)** Dot-blot assays performed with cell wall purified from the same strains.
(TIF)

S6 Fig Absence of Wzd results in smaller cell size. Phase contrast microscopy images of wild-type ATCC6314 strain (WT), its capsule null mutant (BCSMC001; Δ*cps*) and the *wzd* null mutant (BCSMH001; Δ*wzd*) were used to determine cell size. Bacteria were grouped in three different classes depending on their cell cycle stage: (I) recently divided cells; (II) cells initiating division as seen from invagination of cell surface; (III) cells at the final steps of division, with deep invagination at division septum. Lack of Wzd, but not of CPS, results in smaller cells.
(TIF)

S1 Table Results obtained with the statistical analyses of data presented. Analysis of data of the beta-galactosidase activity presented in the different figures was done using a Kruskal-Wallis Test, and a Mann-Whitney Test. P values ≤ 0.05 were considered as significant for all analysis performed.
(XLSX)
