# Supplementary figures and images for: Encapsulation of the septal cell wall protects Streptococcus pneumoniae from its major peptidoglycan hydrolase and host defenses
Source: PLoS Pathog. 2022 Jun 22;18(6):e1010516. doi: 10.1371/journal.ppat.1010516 (PMC9216600; doi:10.1371/journal.ppat.1010516)

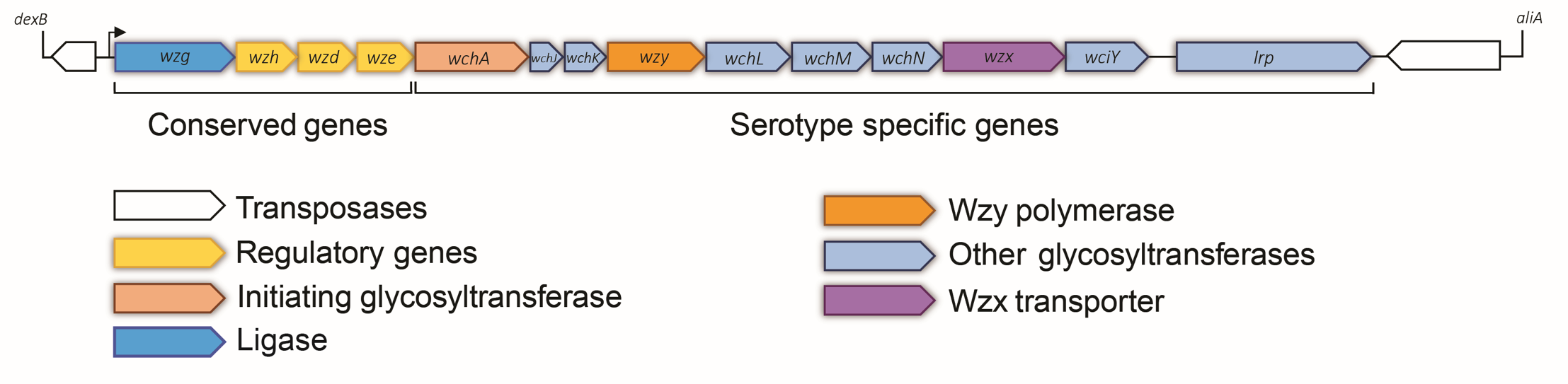

Supplement: S1 Fig — Represented in figure are serotype specific genes that encode several glycosyltransferases that link or modify the different sugars present in the capsule repeating unit (light blue); that attach the first sugar of the repeating unit to the lipid anchor (WchA, light orange); the wzx gene (purple), which encodes the transporter of the CPS repeating unit from the inner to the outer face of the bacterial membrane; the wzy gene (orange), which encodes the polymerase involved in the polymerization of different repeating units and in the assembly of a mature CPS. The first four genes at the 5’ end of the cps operon are highly conserved between serotypes and are proposed to be involved in the regulation of the synthesis of CPS. The first gene is wzg (dark blue), which encodes a ligase capable of attaching the capsule to the peptidoglycan macromolecule. The other three regulatory genes (yellow) encode Wze, an autophosphorylating tyrosine kinase; Wzd, a membrane protein required for the autophosphorylation of Wze and Wzh, a phosphotyrosine protein phosphatase that dephosphorylates Wze. Data adapted from Bentley et al. (14). (TIF) [file ppat.1010516.s001.tif]

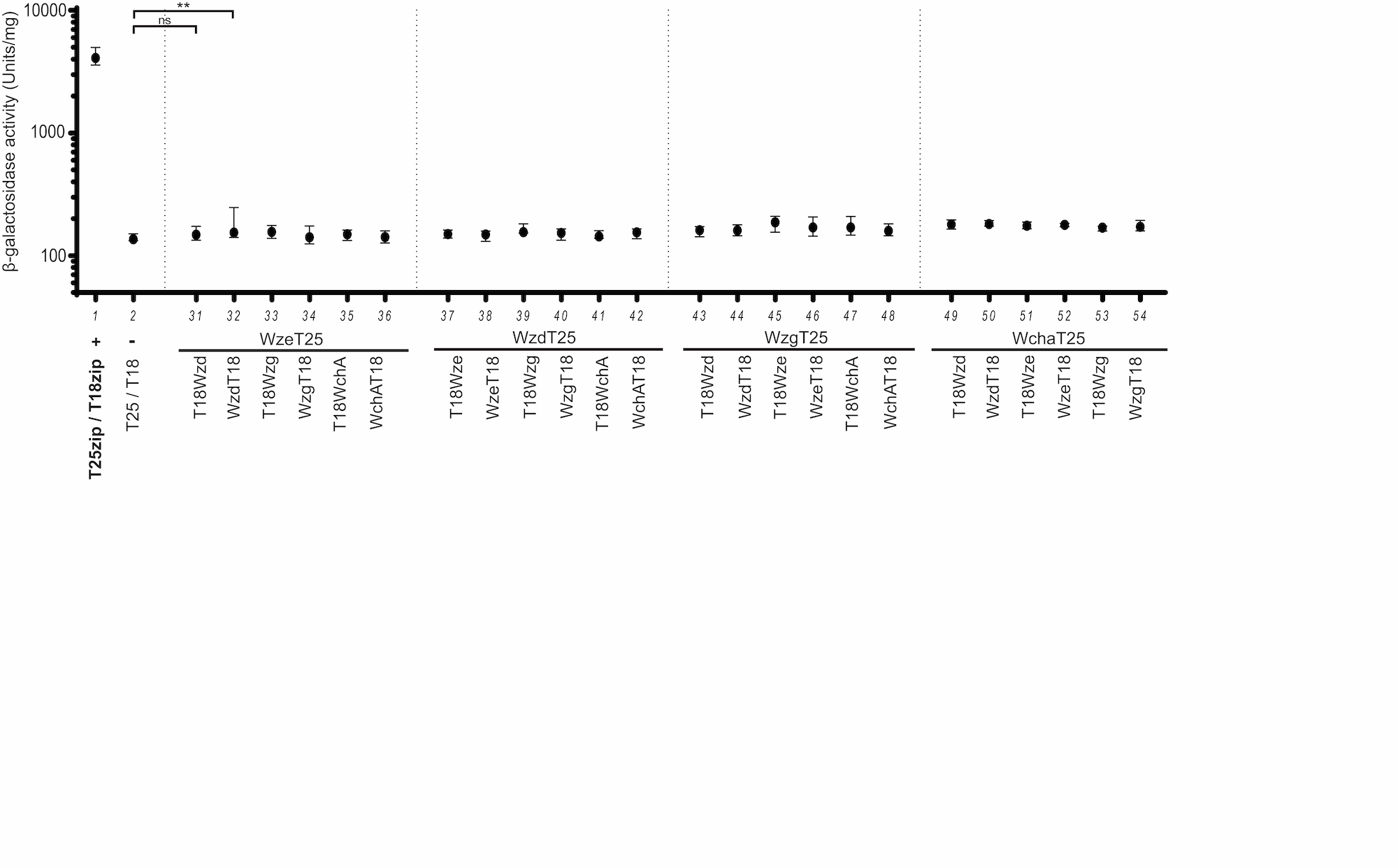

Supplement: S2 Fig — Wze, Wzd, Wzg and WchA interactions were tested using Bacterial Two-Hybrid E. coli system. β-galactosidase activity of cells expressing putative interaction partners was measured in cell extracts in at least three independent replicates. Black circles indicate median values and brackets show the 25% and 75% percentiles. Positive control (+): E. coli expressing T18 and T25 fragments linked to leucine zipper domains (zip) that can dimerize; Negative control (-): E. coli expressing untagged T18 and T25 fragments. No interactions were detected between tested proteins. (TIF) [file ppat.1010516.s002.tif]

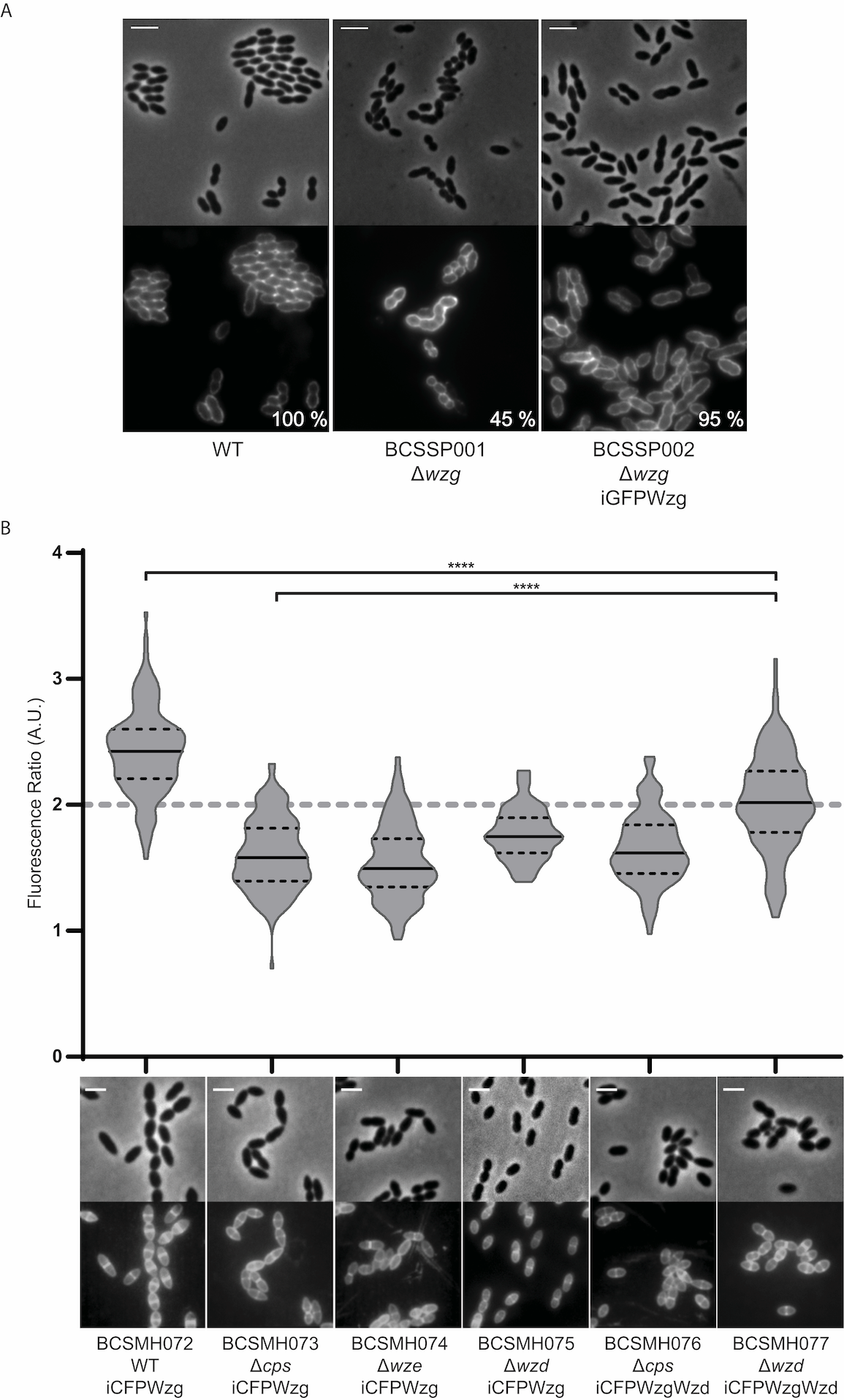

Supplement: S3 Fig — A) Expression of iGFPWzg complements the ability of ATCC6314 wzg null to produce capsule. Immunofluorescence microscopy images using a serotype-14 specific serum to detect the presence of the capsular polysaccharide at the cell surface show that all cells of wild-type encapsulated ATCC6314 strain are surrounded by the capsule over the entire surface and that this number is reduced to 45% in its wzg null mutant strain (BCSSP001). Expression of iGFPWzg encoded in a replicative plasmid in BCSSP002 strain allows the expression of capsule in 95% of bacteria. Representative phase contrast (top panels, for visualization of bacteria) and fluorescence microscopy (middle panels, for detection of the capsule associated with the bacterial cell surface) images of each strain are shown. Scale bar, 2 μm. B) Septal localization of Wzg is dependent on the expression of Wzd/Wze. Graph shows the ratio of iCFP-Wzg fluorescence measured at the septum versus the peripheral wall in the S. pneumoniae wild-type encapsulated strain (BCSMH072, n = 132), the capsule null mutant (BCSMH073, n = 129), the wze null mutant (BCSMH074, n = 130), the wzd null mutant (BCSMH075, n = 112) and in the cps and wzd null mutants expressing Wzd from a constitutive promoter (BCSMH076, n = 135, and BCSMH077, n = 154, respectively). Enrichment of Wzg at the septum is only observed when Wzd is expressed and is localized at the division septum. Solid lines indicate median, and dashed lines indicate 25% and 75% percentiles. Representative phase contrast and fluorescence microscopy images of each strain are shown below the graph. Scale bar, 2 μm. (TIF) [file ppat.1010516.s003.tif]

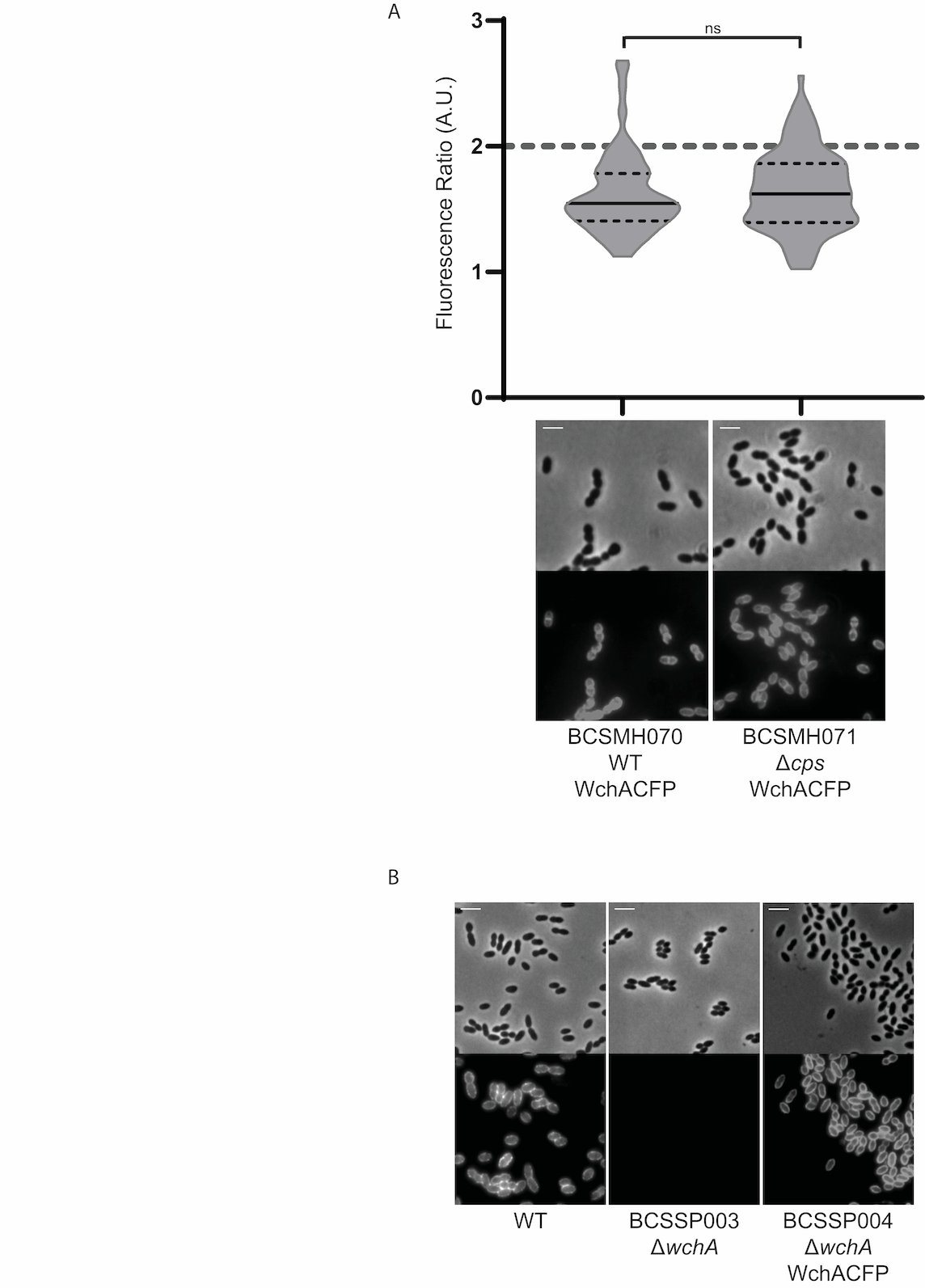

Supplement: S4 Fig — A) Membrane localization of WchA is independent of the presence of the cps operon. Graph shows the ratio of WchA-CFP fluorescence measured at the septum versus the peripheral wall in the S. pneumoniae wild-type encapsulated strain (BCSMH070, n = 101) and in the capsule null mutant (BCSMH071, n = 101). No difference in the localization of WchA was observed between encapsulated and non-encapsulated bacteria. Solid lines indicate median, and dashed lines indicate 25% and 75% percentiles. Representative phase contrast and fluorescence microscopy images of each strain are shown below the graph. Scale bar, 2 μm. B) Expression of WchACFP complements the ability of ATCC6314 wchA null to produce capsule. Immunofluorescence microscopy images using a serotype-14 specific serum to detect the presence of the capsular polysaccharide at the cell surface show that all cells of wild-type encapsulated ATCC6314 strain are surrounded by the capsule over the entire surface and that no cells expressing capsule can be observed in its wchA null mutant strain (BCSSP003). Expression of WchACFP encoded in a replicative plasmid in BCSSP004 strain allows the expression of capsule in most bacteria. Representative phase contrast (top panels, for visualization of bacteria) and fluorescence microscopy (middle panels, for detection of the capsule associated with the bacterial cell surface) images of each strain are shown. Scale bar, 2 μm. (TIF) [file ppat.1010516.s004.tif]

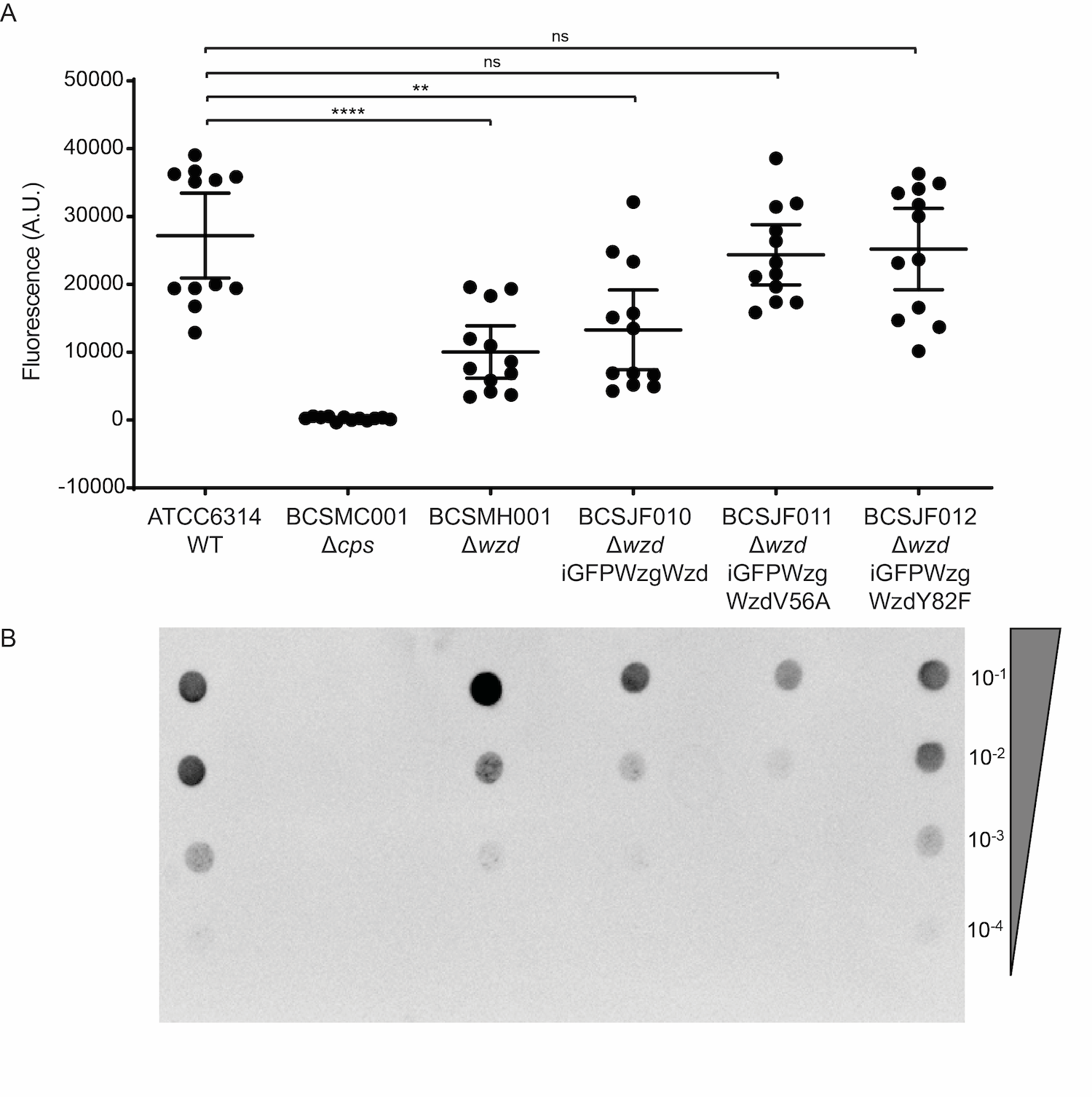

Supplement: S5 Fig — A) Cells from exponentially growing cultures of ATCC6314 (encapsulated parental strain, WT), BCSMC001 (non-encapsulated Δcps mutant strain), BCSMH001 (Δwzd mutant strain) and its derivatives strains that carry a plasmid expressing iGFPWzg in the presence of Wzd (BCSJF010 strain); of the mutated WzdV56A protein (BCSJF011 strain) and of the mutated WzdY82F protein (BCSJF012 strain) were analysed by dotblot using serotype-14 specific serum. Graph shows the intensity of the fluorescence signal measured in dot-blot assays in three independent experiments. Expression of WzdY82F and WzdV56A in the wzd null mutant strain allows expression of capsule at levels similar to those observed with the parental strain. B) Dot-blot assays performed with cell wall purified from the same strains. (TIF) [file ppat.1010516.s005.tif]

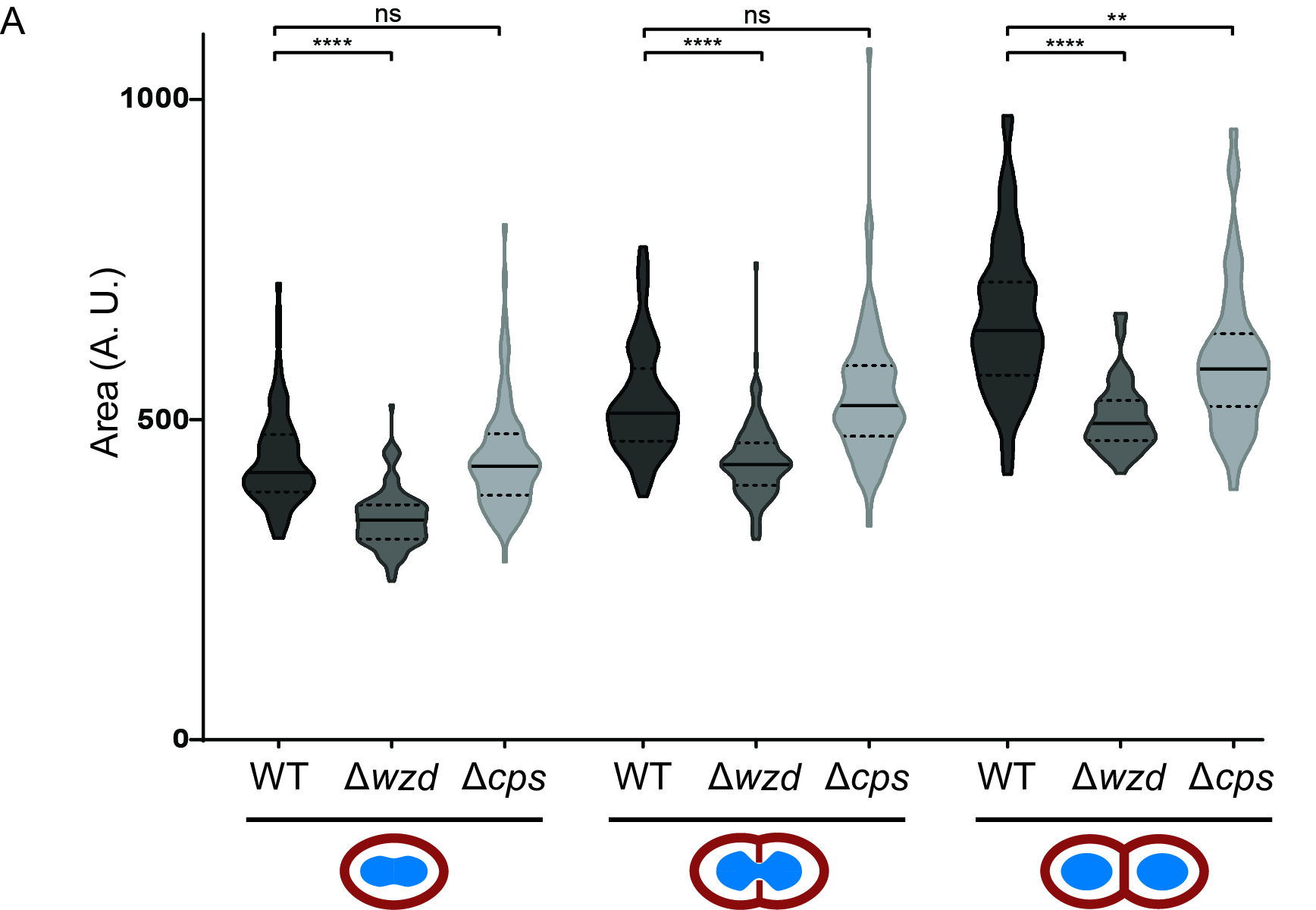

Supplement: S6 Fig — Phase contrast microscopy images of wild-type ATCC6314 strain (WT), its capsule null mutant (BCSMC001; Δcps) and the wzd null mutant (BCSMH001; Δwzd) were used to determine cell size. Bacteria were grouped in three different classes depending on their cell cycle stage: (I) recently divided cells; (II) cells initiating division as seen from invagination of cell surface; (III) cells at the final steps of division, with deep invagination at division septum. Lack of Wzd, but not of CPS, results in smaller cells. (TIF) [file ppat.1010516.s006.tif]
